# Supplementary material for: Robust phenotyping of highly multiplexed tissue imaging data using pixel-level clustering
Source: Nat Commun. 2023 Aug 1;14:4618. doi: 10.1038/s41467-023-40068-5 (PMC10393943; doi:10.1038/s41467-023-40068-5)
Supplement: Supplementary file 1 — Supplementary Information [file 41467_2023_40068_MOESM1_ESM.pdf]

Supplementary Information

Liu et al., Robust phenotyping of highly multiplexed tissue imaging data using pixel-level clustering

Supplementary Figure 1: Challenges with analyzing multiplexed imaging data

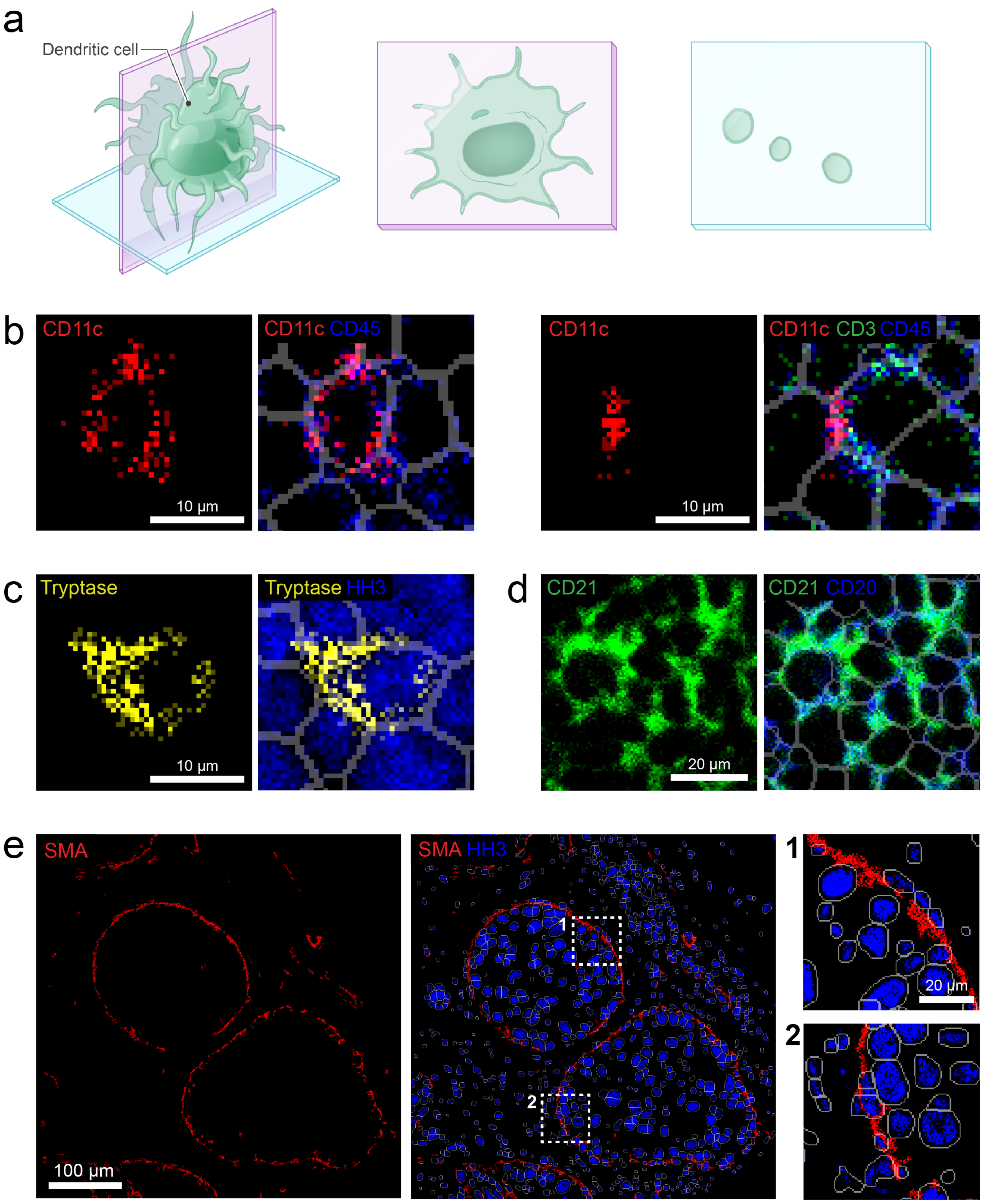

(A) Illustration of sectioning a 3D object in 2D. The objects that are viewed on the slide are highly dependent on the sectioning plane. (B) Example of a dendritic cell marker, CD11c, appearing cellular (left) or as an acellular object (right).

Gray lines correspond to cell boundaries obtained using segmentation. (C) Example of a marker overlapping into the neighboring cell. A mast cell marker (tryptase) is shown here. (D) Example of two overlapping markers when tissue is dense, and cells are in close contact with each other. A follicular dendritic cell and B cell marker (CD21) and B cell marker (CD20) are shown here. (E) Example of a feature not captured by traditional cell segmentation. The thin myoepithelial layer surrounding the ductal cells in ductal carcinoma in situ (DCIS) is shown here.

Supplementary Figure 2: Pixel clustering pipeline in Pixie

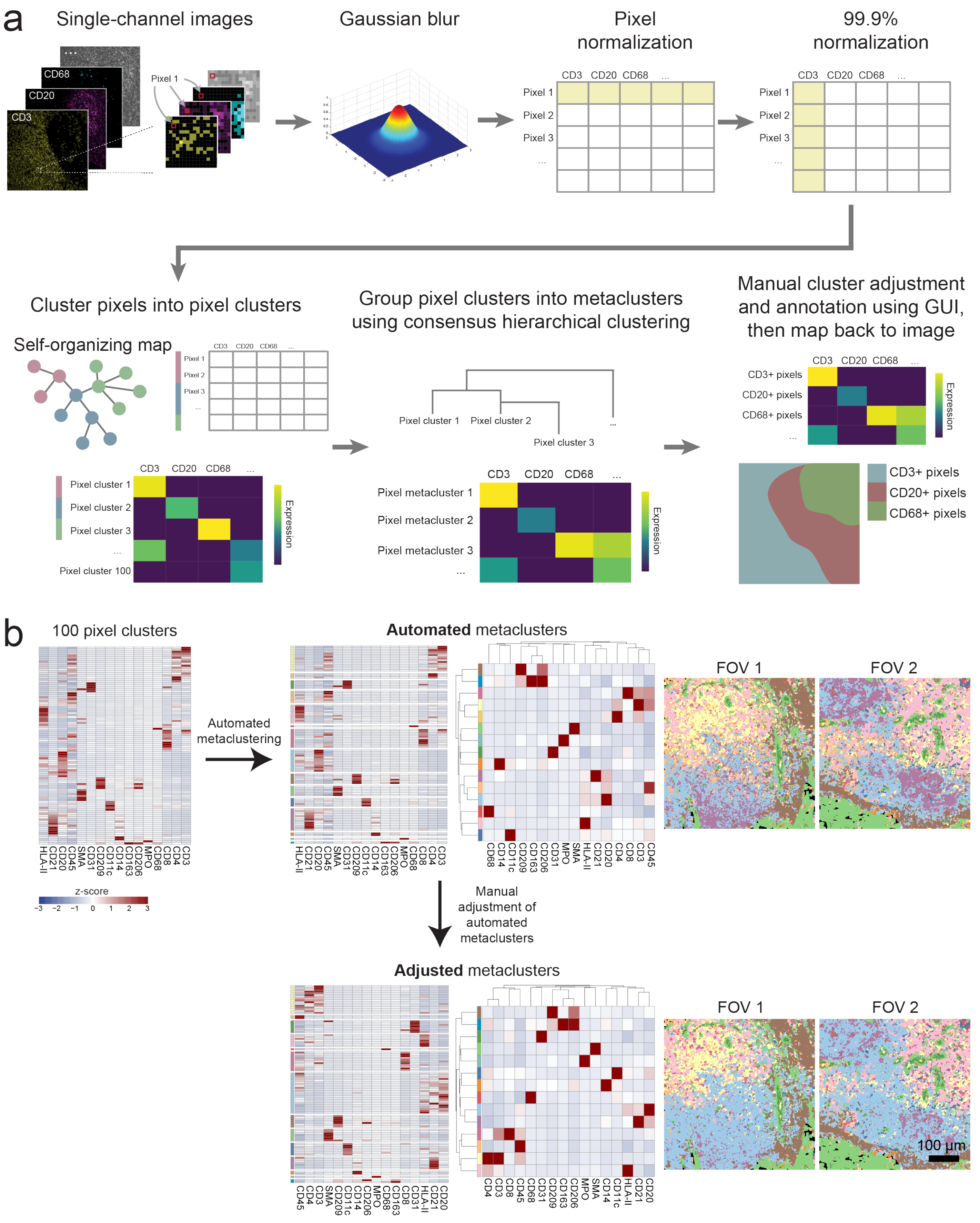

(A) Detailed overview of the pixel clustering pipeline in Pixie. First, pixel expression profiles are extracted from the images. A Gaussian blur, pixel-level normalization, and 99.9% marker normalization are applied. The transformed pixels are then clustered using a self-organizing map (SOM). The clusters output by the SOM are metaclustered using consensus hierarchical clustering. Finally, the user can manually adjust the metaclusters and annotate each metacluster with its phenotype based on its expression profile using our easy-to-use GUI. These final pixel clusters can then be mapped back to the original images. (B) Comparison of manual metacluster adjustment. After SOM clustering, the 100 clusters are metaclustered using consensus hierarchical clustering, which is a fully automated process (top). The assignment of clusters to the metaclusters can then be manually adjusted to better reflect expected biology (bottom). Pixel phenotype maps for automated metaclusters and adjusted metaclusters are shown (right).

Supplementary Figure 3: Additional examples of pixel clustering

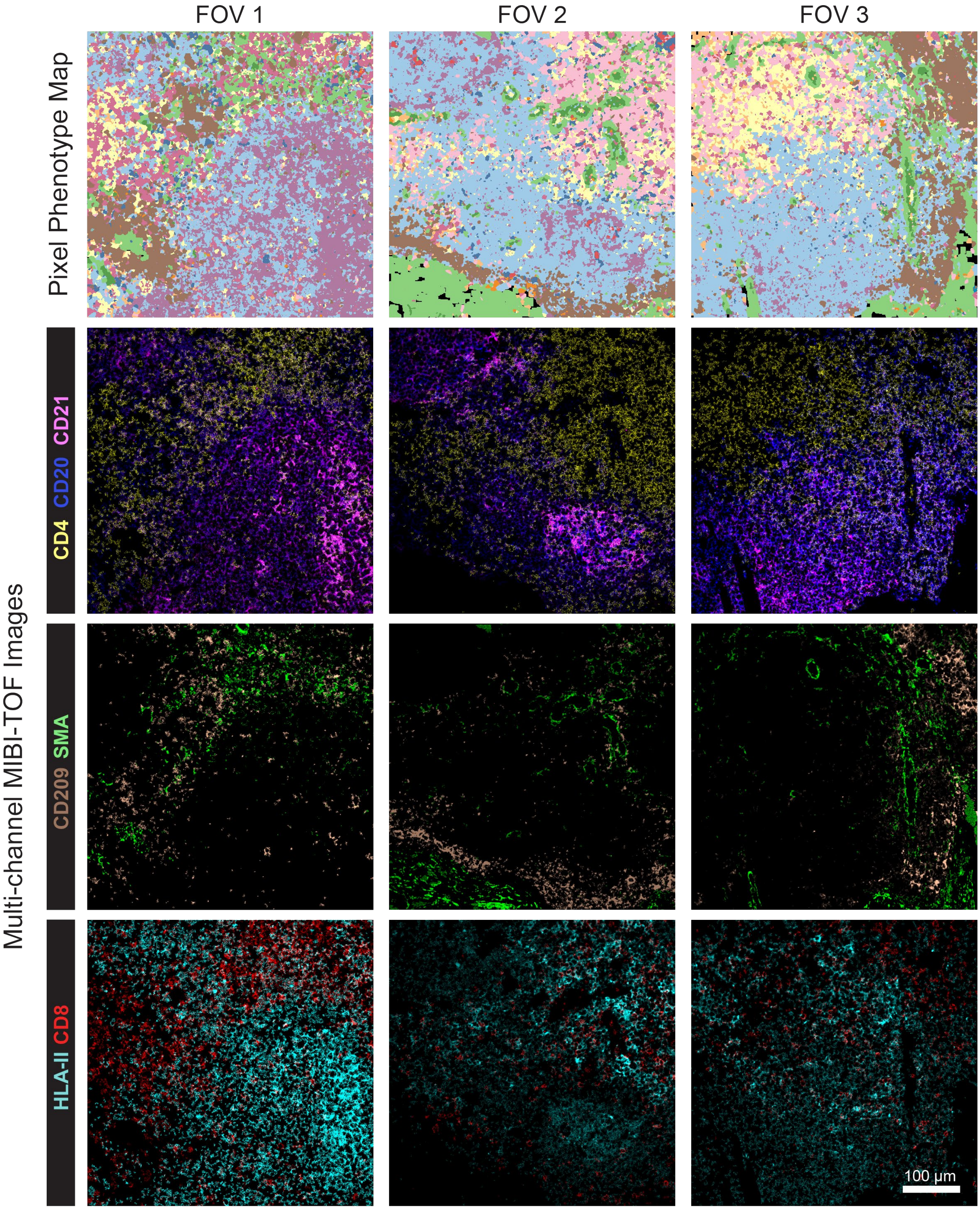

Additional examples of pixel clustering of the lymph node dataset. Top row shows the pixel phenotype maps, where the colors correspond to the heatmap in Fig. 2b. The second, third, and fourth rows show MIBI-TOF overlays for various markers. Each column is one FOV.

Supplementary Figure 4: Assessing reproducibility

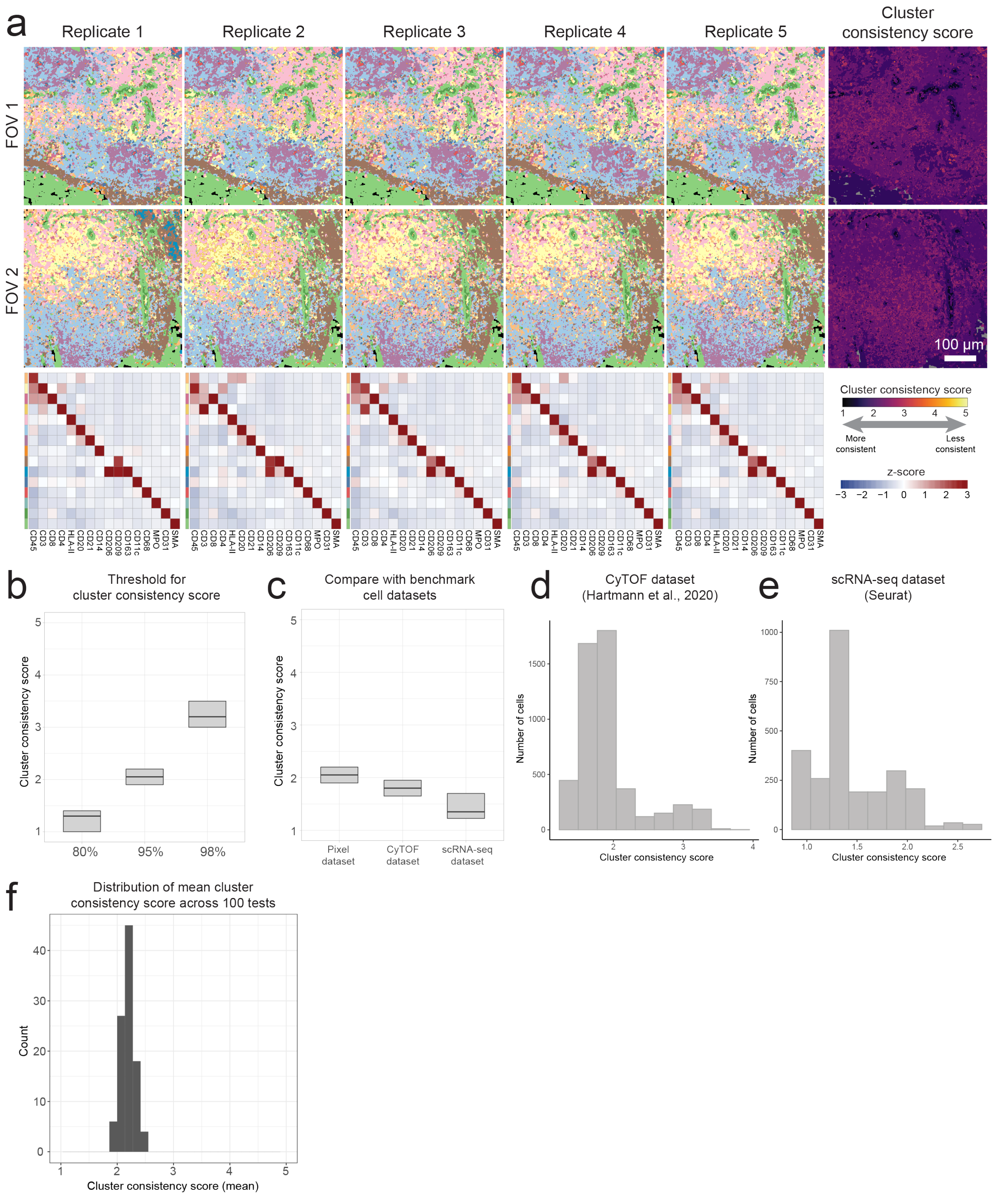

(A) Pixel phenotype maps of five replicates of the same FOV (each replicate initialized with a different seed). Pixel phenotype maps colored according to the heatmaps in the bottom row. The right column shows the same FOV colored according to

cluster consistency score. (B) Distribution of cluster consistency score across all pixels for different thresholds for calculating the cluster consistency score. Boxplots show median as the center and 25th and 75th percentiles as the bounds of the box. n=12,515,748 pixels from 12 images. (C) Comparison of cluster consistency score distribution of the lymph node dataset (all pre-processing steps) and two benchmark cell datasets, a reference CyTOF dataset and single-cell RNA-sequencing dataset. Boxplots show median as the center and 25th and 75th percentiles as the bounds of the box. Pixel dataset: n=12,515,748 pixels from 12 images, CyTOF dataset: n=1,140,035 cells, scRNA-seq dataset: n=2,700 cells. (D) Distribution of cluster consistency score for a reference CyTOF dataset.[61] (E) Distribution of cluster consistency score for a reference single-cell RNA-sequencing dataset (2,700 PBMC dataset from the Seurat tutorial website).[62] (F) Distribution of mean cluster consistency score across 100 tests, where each test comprised of 5 replicates of the SOM trained on the same dataset.

Supplementary Figure 5: Gaussian blur

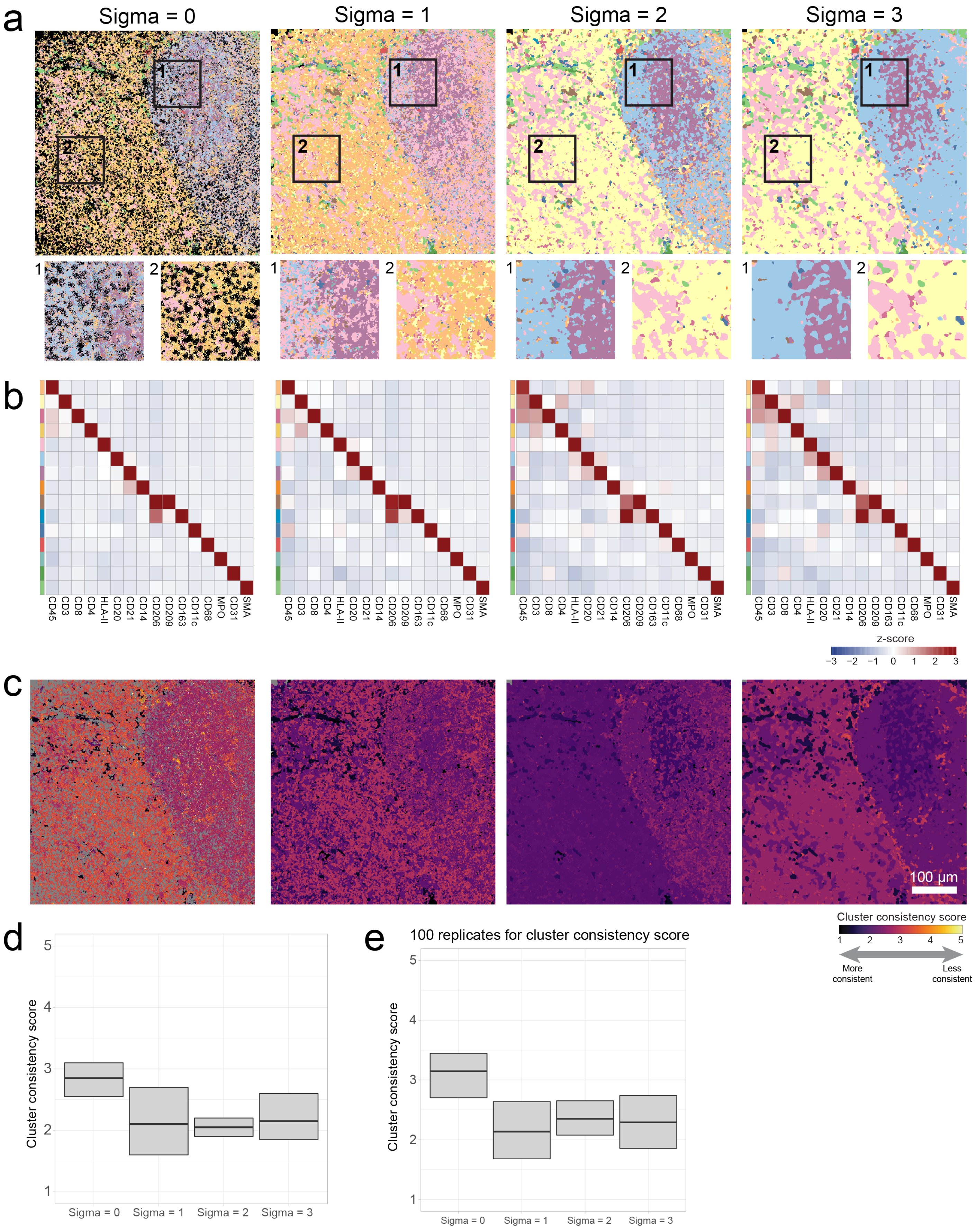

(A) Pixel phenotype maps showing the effect of increasing the standard deviation (sigma) for the Gaussian kernel of the blur. (B) Heatmaps of the pixel cluster expression profiles corresponding to each sigma. Expression values were z-scored for each marker. Colors of the color bars correspond to the pixel phenotype maps in A. (C) FOVs colored according to cluster consistency score for each sigma. (D) Comparison of the distribution of cluster consistency scores across all pixels in the dataset for each sigma. Boxplots show median as the center and 25th and 75th percentiles as the bounds of the box. n=12,515,748 pixels from 12 images. (E) Comparison of the distribution of cluster consistency scores across all pixels where 100 replicates were used for the cluster consistency score calculation. Boxplots show median as the center and 25th and 75th percentiles as the bounds of the box. n=12,515,748 pixels from 12 images.

Supplementary Figure 6: No pixel normalization

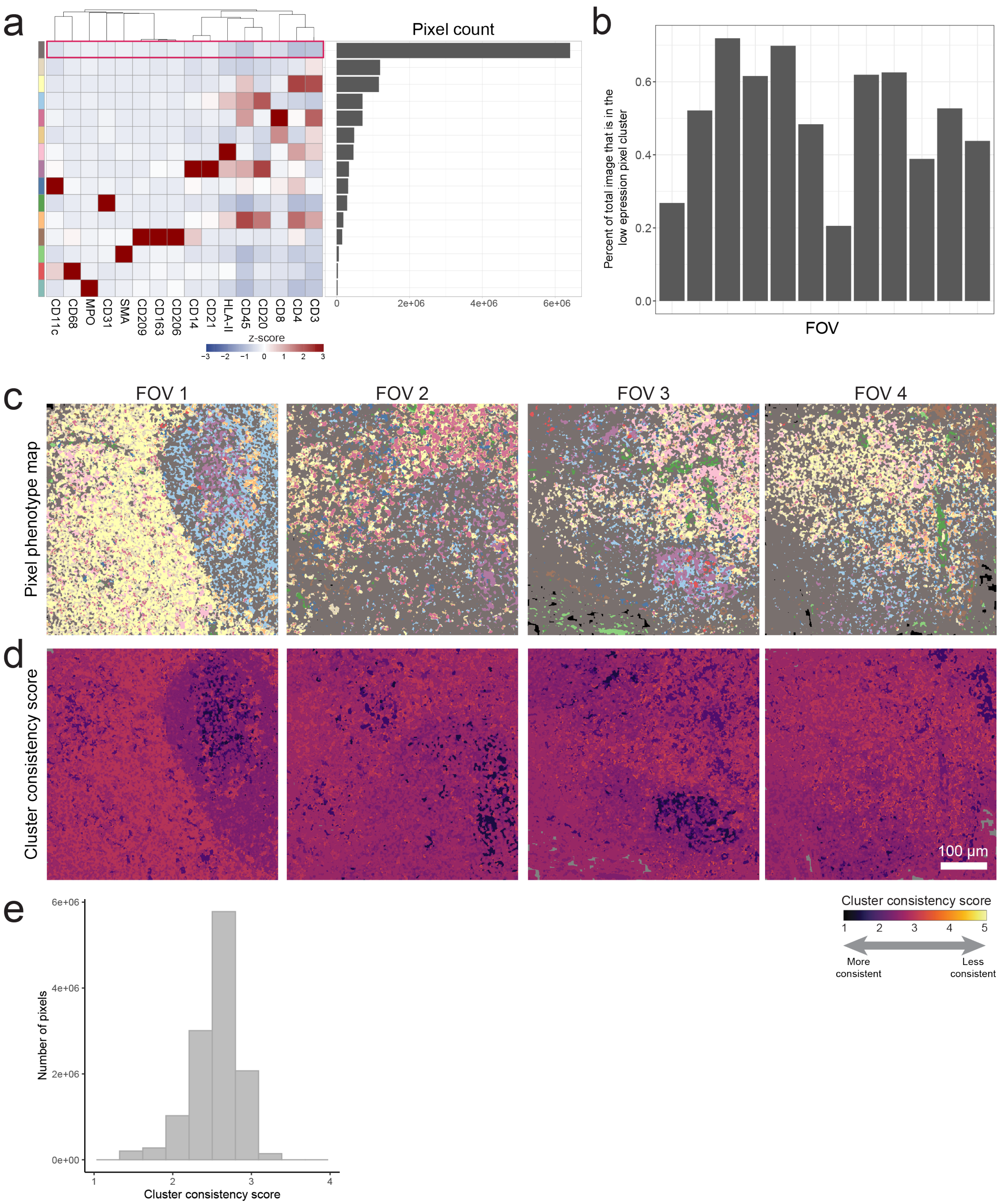

(A) Heatmap of mean marker expression of pixel cluster phenotypes where there was no pixel normalization performed. Expression values were z-scored for each marker. The number of pixels per cluster is shown on the right. (B) The

percentage of the total pixels in each image that were assigned to the low expression pixel cluster. (C) Pixel phenotype maps for four representative FOVs (colored according to the heatmap in A). (D) The same FOVs in C colored according to cluster consistency score. (E) The distribution of cluster consistency score across all pixels in the dataset.

Supplementary Figure 7: No pixel normalization in additional datasets

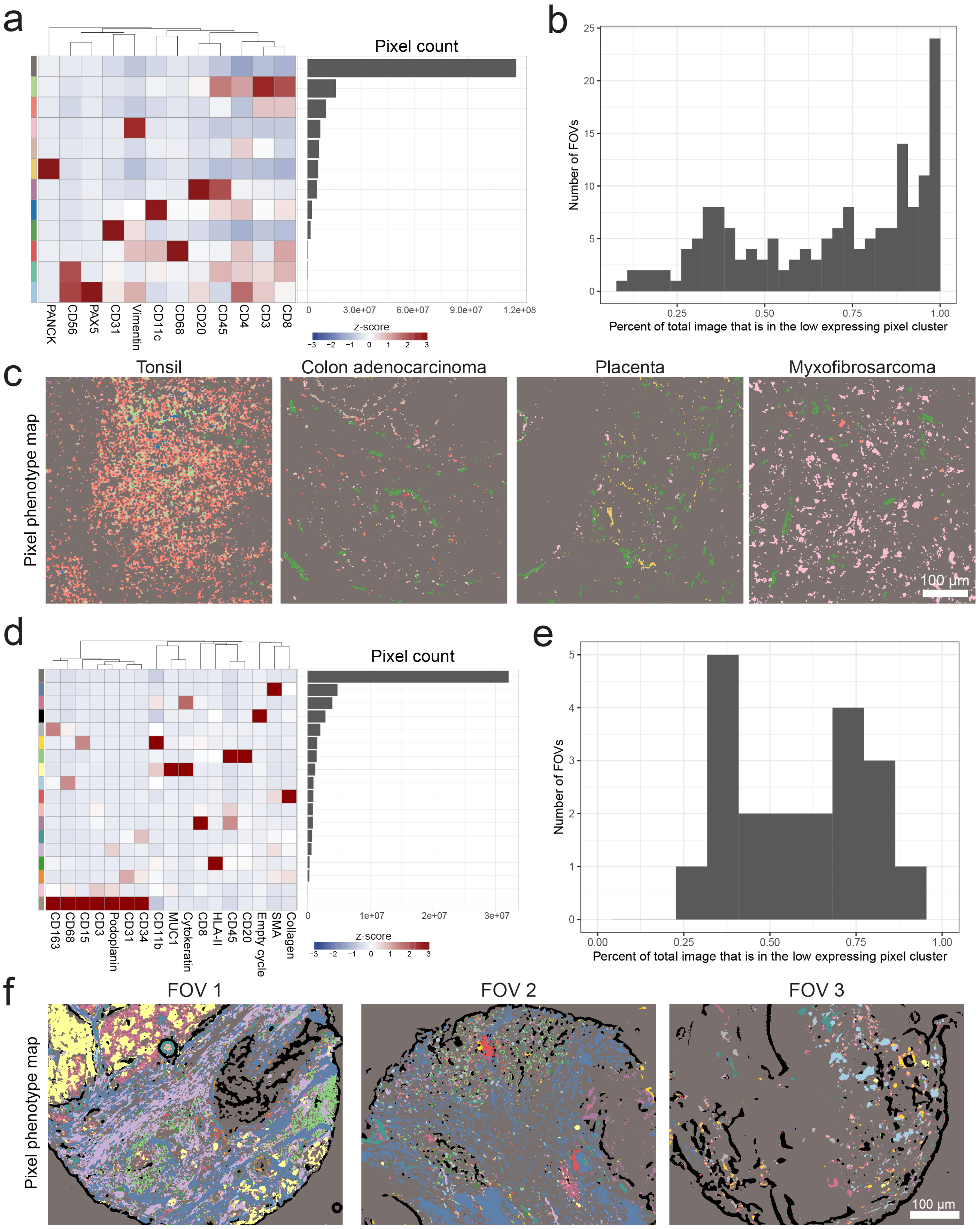

(A-C) Pixel clustering with no pixel normalization applied to MIBI-TOF dataset of 21 different tissue cores.[35] (D-F) Pixel clustering with no pixel normalization applied to CODEX dataset of colorectal cancer.[17] (A, D) Heatmap of mean marker expression of pixel cluster phenotypes where there was no pixel normalization performed. Expression values were z-scored for each marker. The number of pixels per cluster is shown on the right. (B, E) The distribution of the percentage of the total pixels in each image that were assigned to the low expression pixel cluster. (C, F) Pixel phenotype maps for representative FOVs (colored according to the heatmap in A or D, respectively).

Supplementary Figure 8: No 99.9% normalization

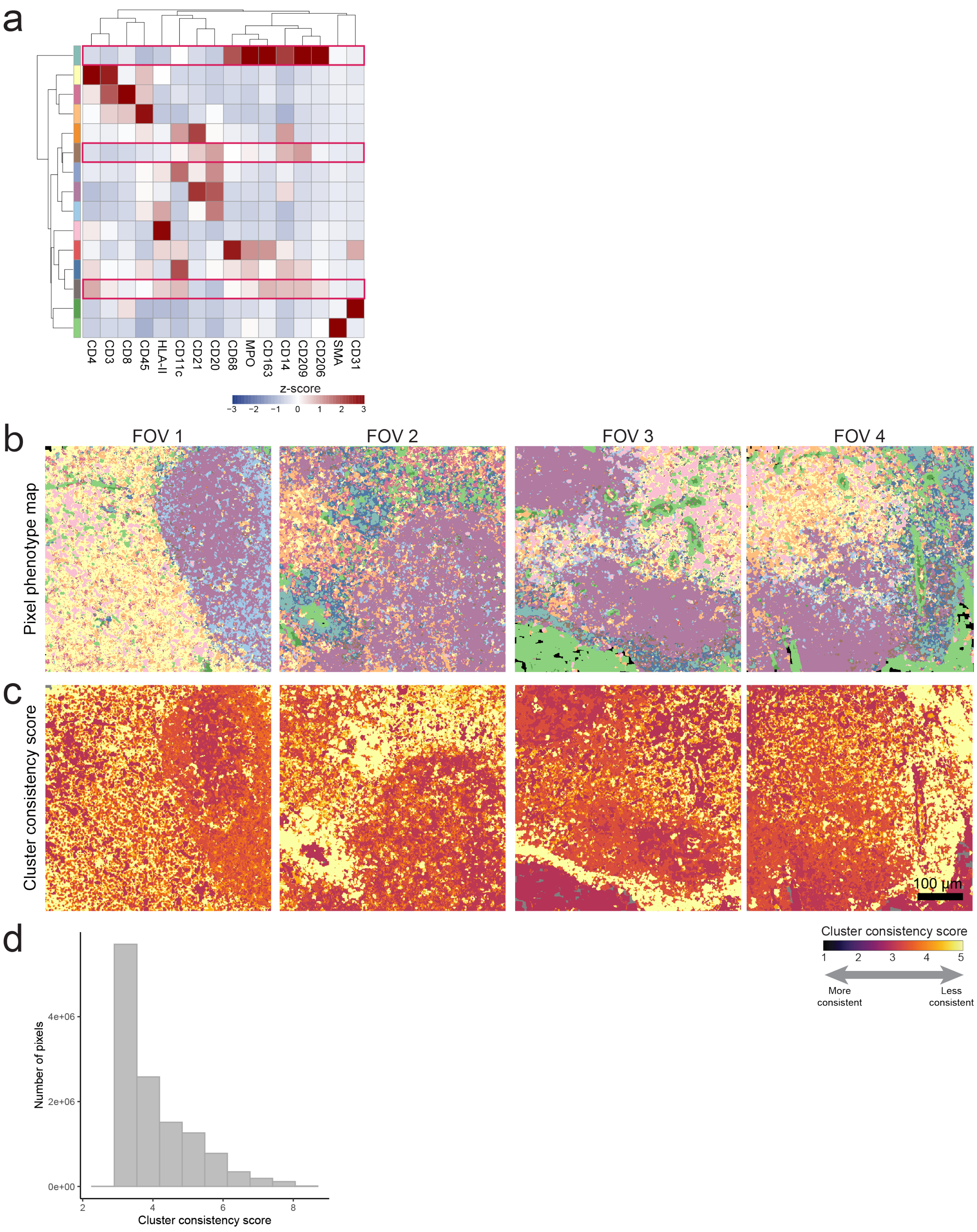

(A) Heatmap of mean marker expression of pixel cluster phenotypes where there was no 99.9% marker normalization performed. Expression values were z-scored for each marker. The red boxes indicate ambiguous pixel clusters with poor cluster definition. (B) Pixel phenotype maps for four representative FOVs (colored according to the heatmap in A). (C) The same FOVs in B colored according to cluster consistency score. (D) The distribution of cluster consistency score across all pixels in the dataset.

Supplementary Figure 9: SOM with 15 nodes

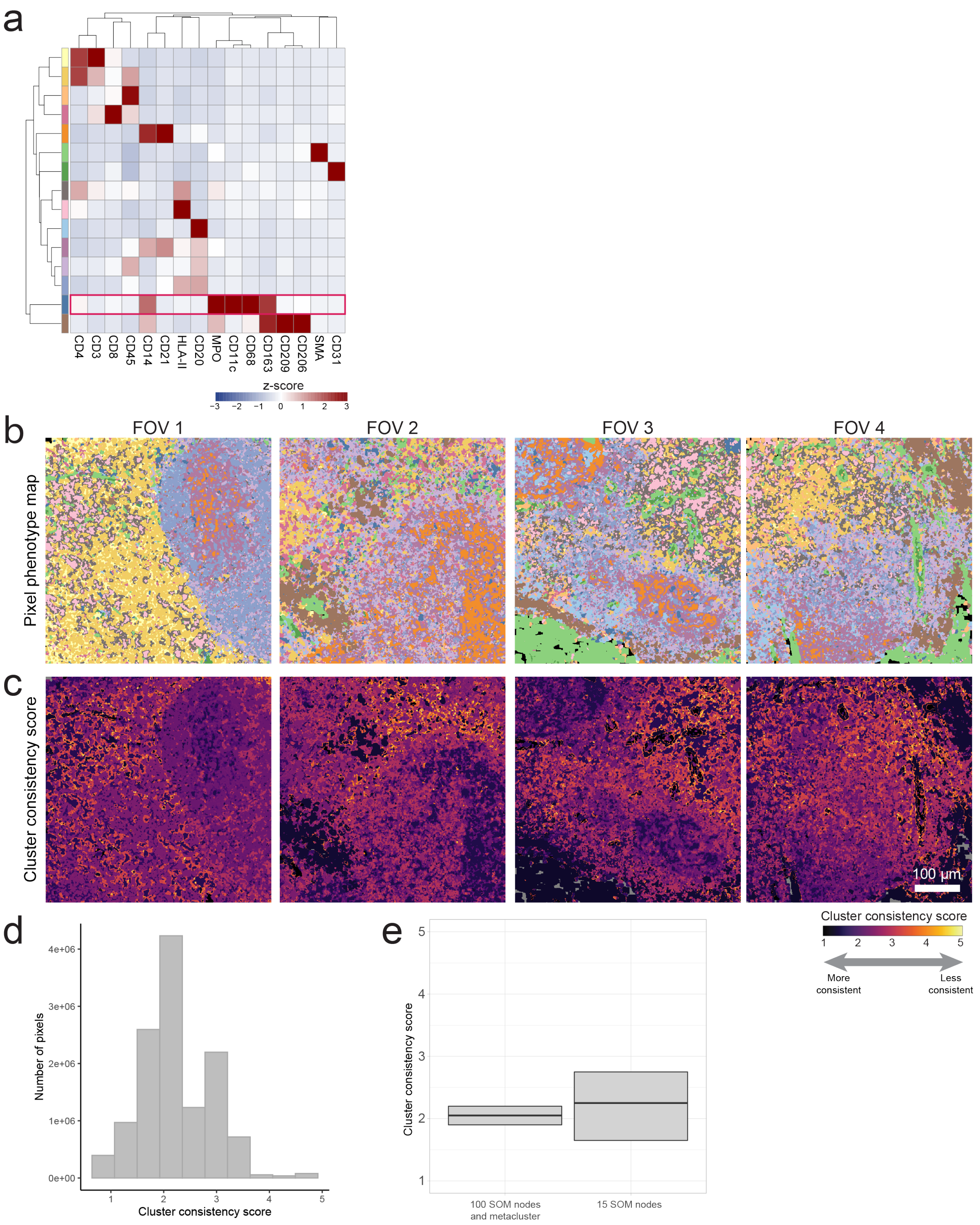

(A) Heatmap of mean marker expression of pixel cluster phenotypes where a SOM was used to cluster pixels directly into 15 clusters. Expression values were z-scored for each marker. The red box indicates an ambiguous pixel cluster with poor cluster definition. (B) Pixel phenotype maps for four representative FOVs (colored according to the heatmap in A). (C) The same FOVs in B colored according to cluster consistency score. (D) The distribution of cluster consistency score across all pixels in the dataset. (E) Comparison of the distribution of cluster consistency scores across all pixels in the dataset for metaclustering vs. directly clustering into 15 clusters. Boxplots show median as the center and 25th and 75th percentiles as the bounds of the box. n=12,515,748 pixels from 12 images.

Supplementary Figure 10: Number of passes

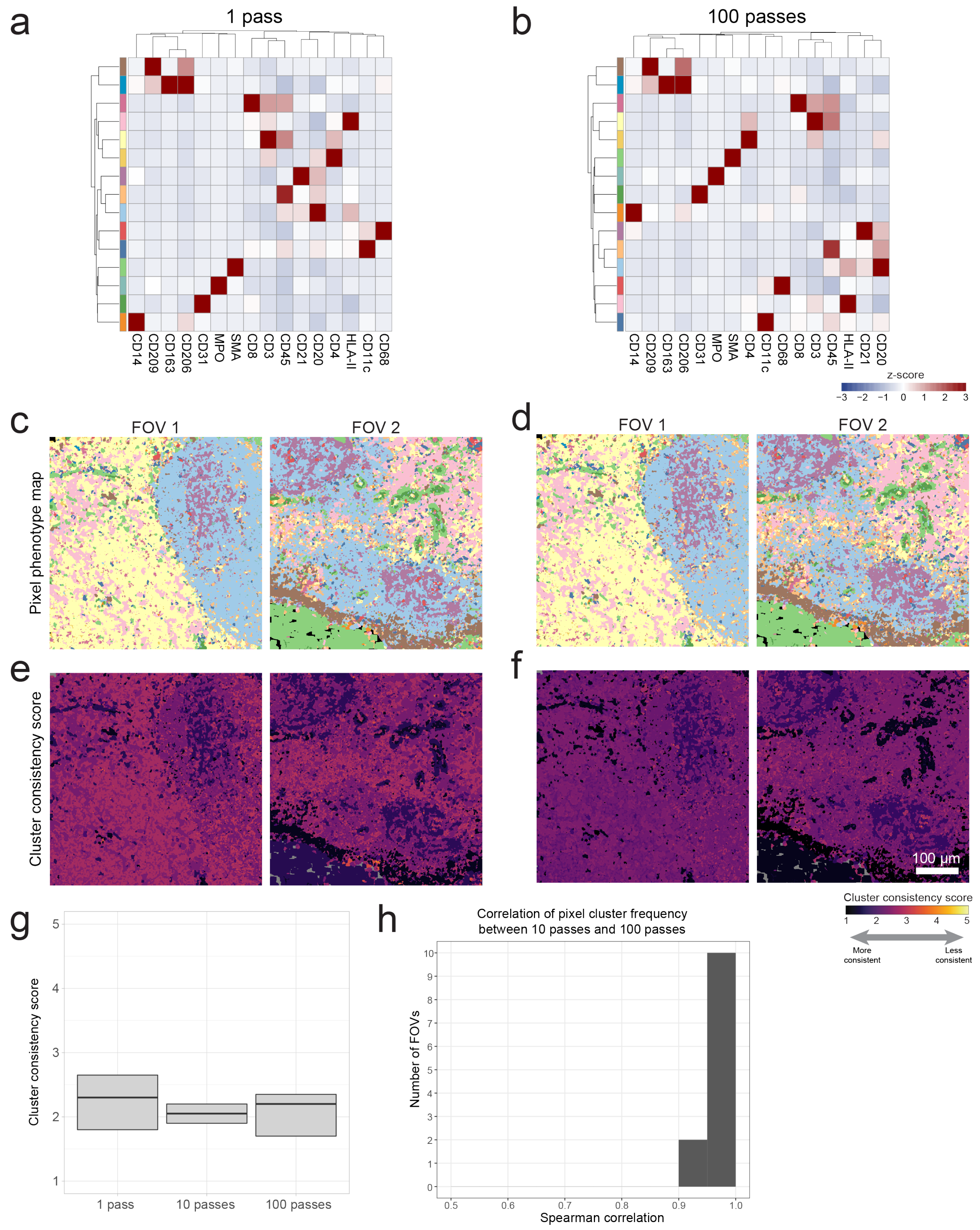

Heatmap of mean marker expression of pixel cluster phenotypes where the SOM was trained using 1 pass (A) or 100 passes (B). Expression values were z-scored for each marker. Pixel phenotype maps for two representative FOVs corresponding to 1 pass (C) or 10 passes (D). Pixel phenotype maps are colored according to A and B, respectively. The same FOVs in C and D colored according to cluster consistency score for 1 pass (E) or 10 passes (F). (G) Comparison of the distribution of cluster consistency scores across all pixels in the dataset for different number of training passes through the SOM. Boxplots show median as the center and 25th and 75th percentiles as the bounds of the box. n=12,515,748 pixels from 12 images. (H) Spearman correlation of pixel cluster frequency between 10 passes and 100 passes.

Supplementary Figure 11: Subset pixels (decidua dataset)

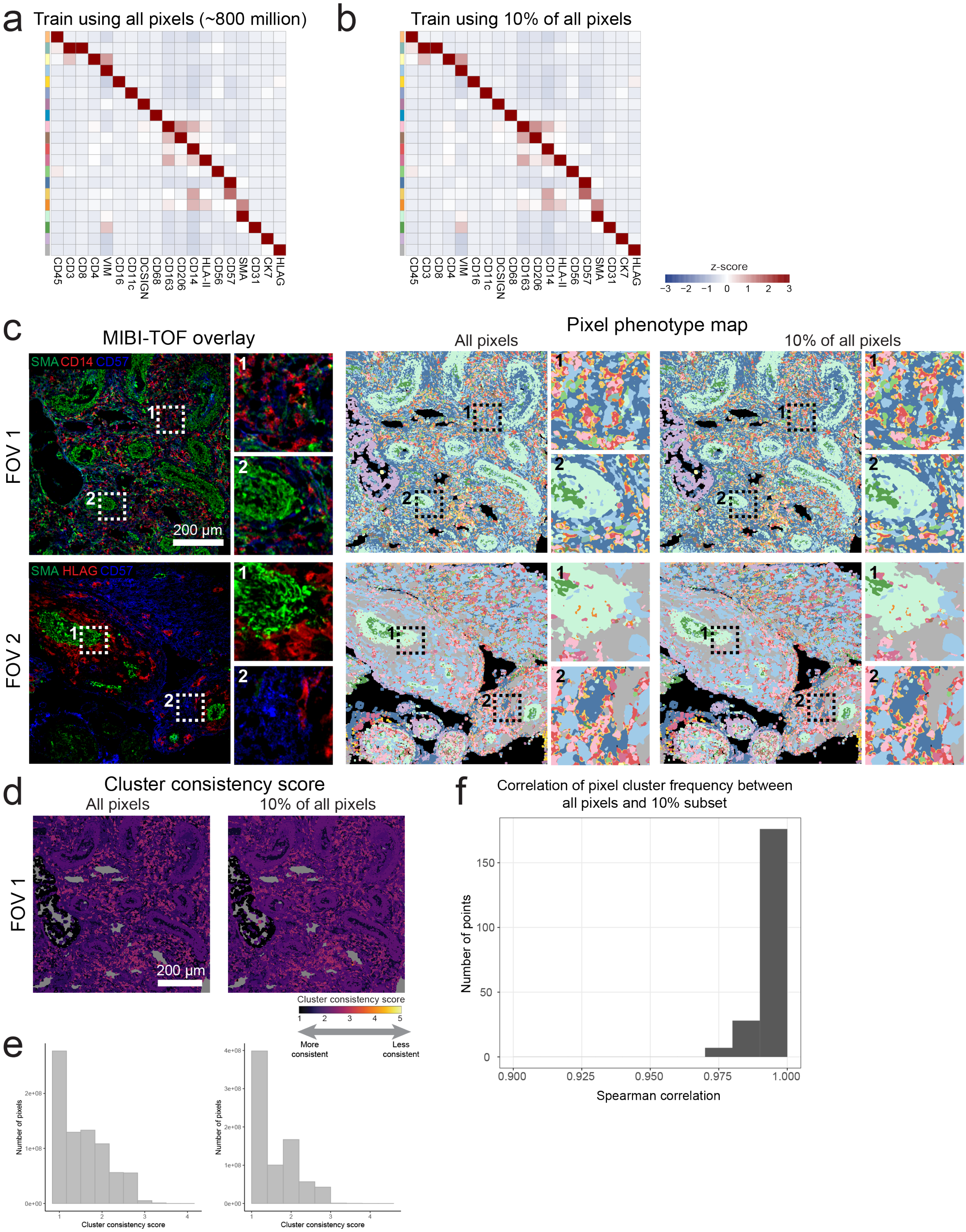

Heatmap of mean marker expression of pixel cluster phenotypes for a dataset of human decidua[33], where the SOM was trained using all pixels (A) or using a 10% subset of pixels (B). Expression values were z-scored for each marker. The full dataset contained a total of 766,440,566 pixels. (C) MIBI-TOF overlays (left) and pixel phenotype maps (right) for two representative FOVs. (D) FOV colored according to cluster consistency score for the SOM trained using all pixels (left) or a subset of pixels (right). (E) Comparison of the distribution of cluster consistency score across all pixels in the dataset for the SOM trained using all pixels (left) or a subset of pixels (right). (F) Spearman correlation of pixel cluster frequency between training using all pixels or a subset of pixels.

Supplementary Figure 12: CyCIF whole slide tonsil dataset

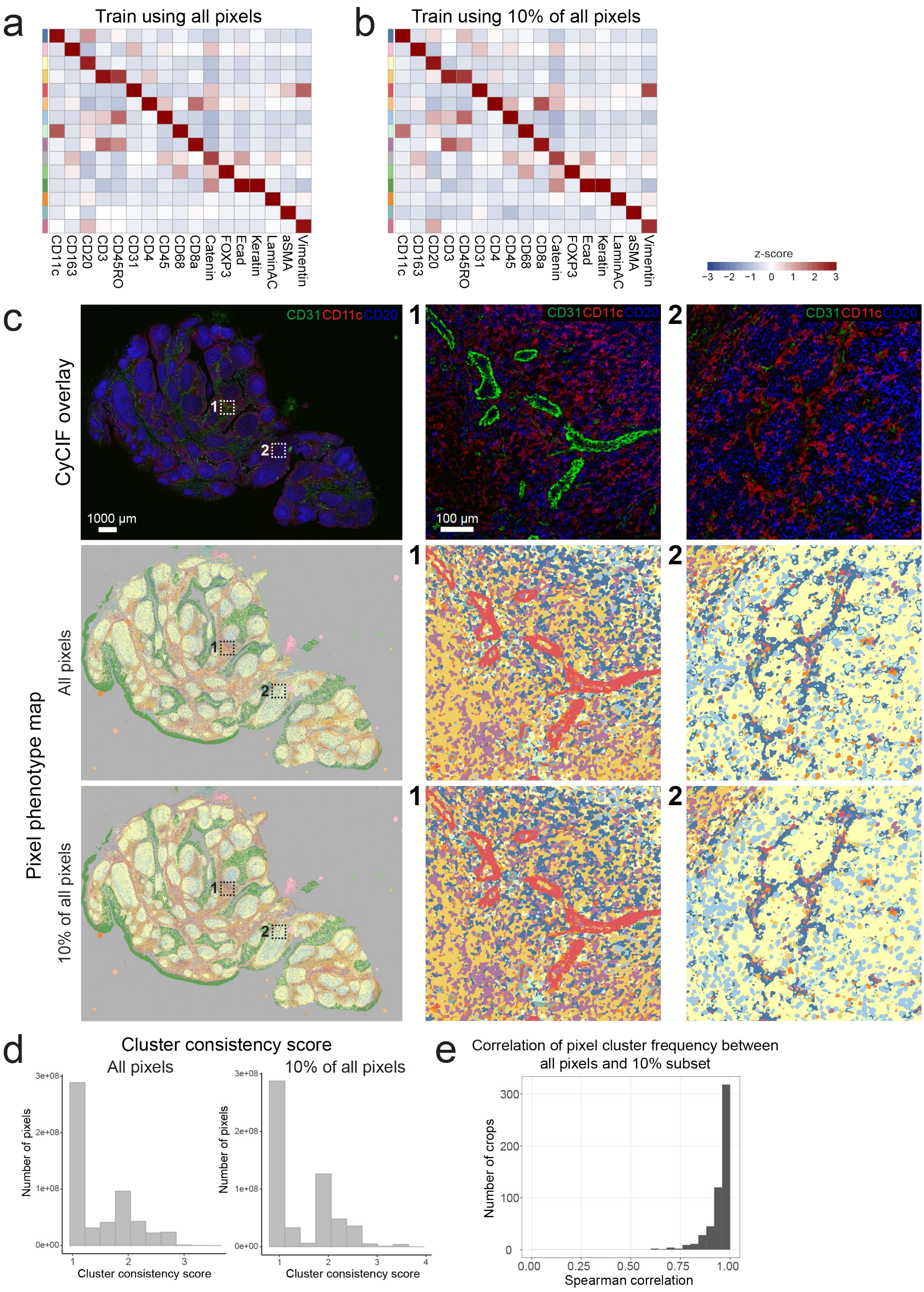

Heatmap of mean marker expression of pixel cluster phenotypes for a whole-slide CyCIF dataset of tonsil tissue[34], where the SOM was trained using all pixels (A) or using a 10% subset of pixels (B). Expression values were z-scored for each marker. The whole slide image was 27,299 x 20,045 pixels. (C) CyCIF overlays (top row) and pixel phenotype maps where the SOM was trained using all pixels (middle row) or using a 10% subset of pixels (bottom row). (D) Distribution of cluster consistency score across all pixels in the dataset for the SOM trained using all pixels (left) or a subset of pixels (right). (E) Spearman correlation of pixel cluster frequency between training using all pixels or a subset of pixels. To match the Spearman correlation calculation of the datasets with individual FOVs, 1024 x 1024 pixel crops were taken of the whole-slide image for the correlation calculation.

Supplementary Figure 13: DCIS and TNBC pixel cluster profiles and TNBC quantification

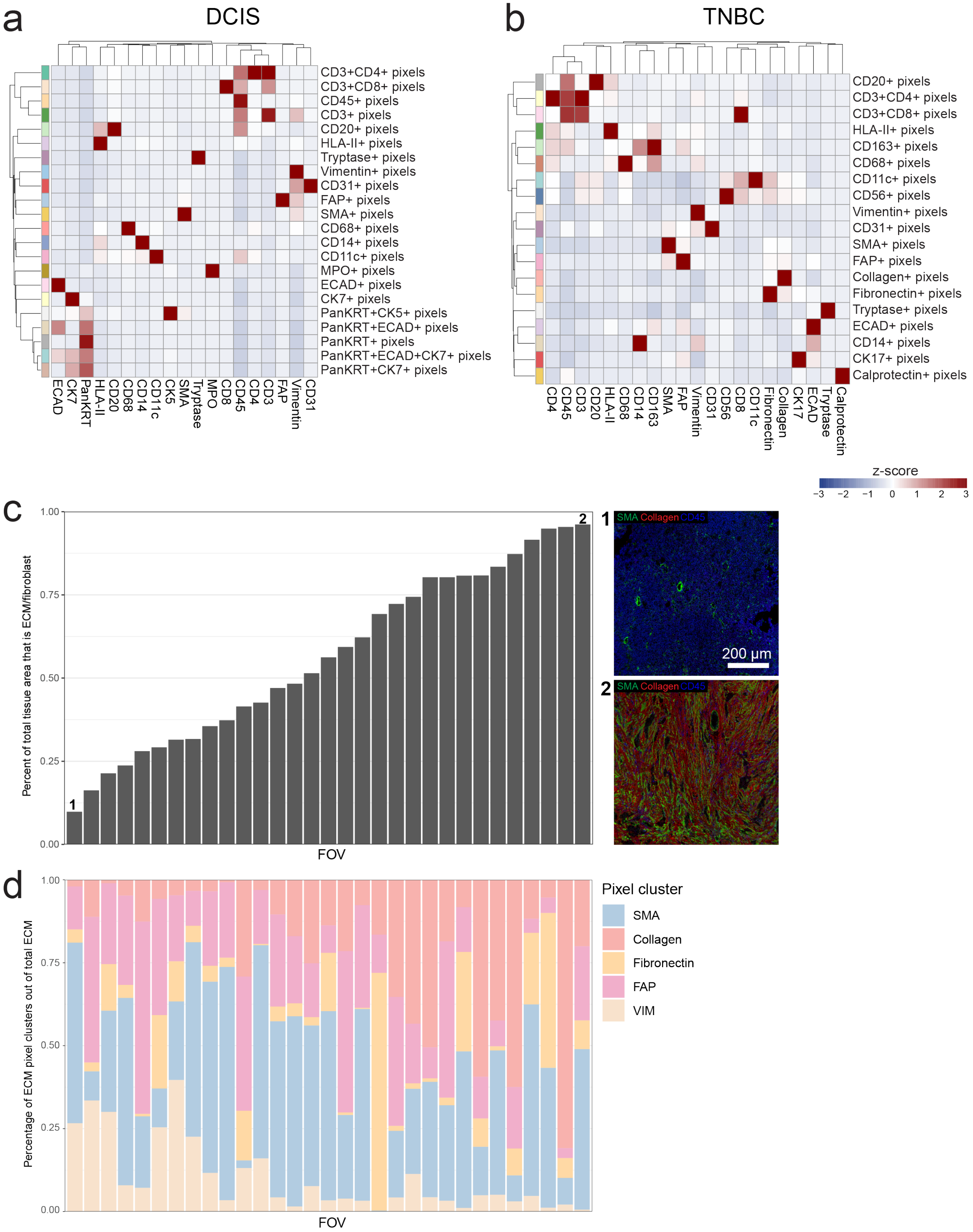

Pixel cluster expression profiles corresponding to the pixel clusters in Fig. 3b (DCIS) and Fig. 3c (TNBC) respectively. Expression values were z-scored for each marker. (C) For each FOV in the TNBC dataset, quantification of the percent of total tissue area that is comprised of pixel clusters of the ECM or fibroblast phenotypes. On the right, MIBI-TOF overlays of the FOVs with the lowest and highest amount of ECM/fibroblast. (D) Breakdown of the ECM/fibroblast pixel clusters for each FOV.

Supplementary Figure 14: Reproducibility of pixel clustering

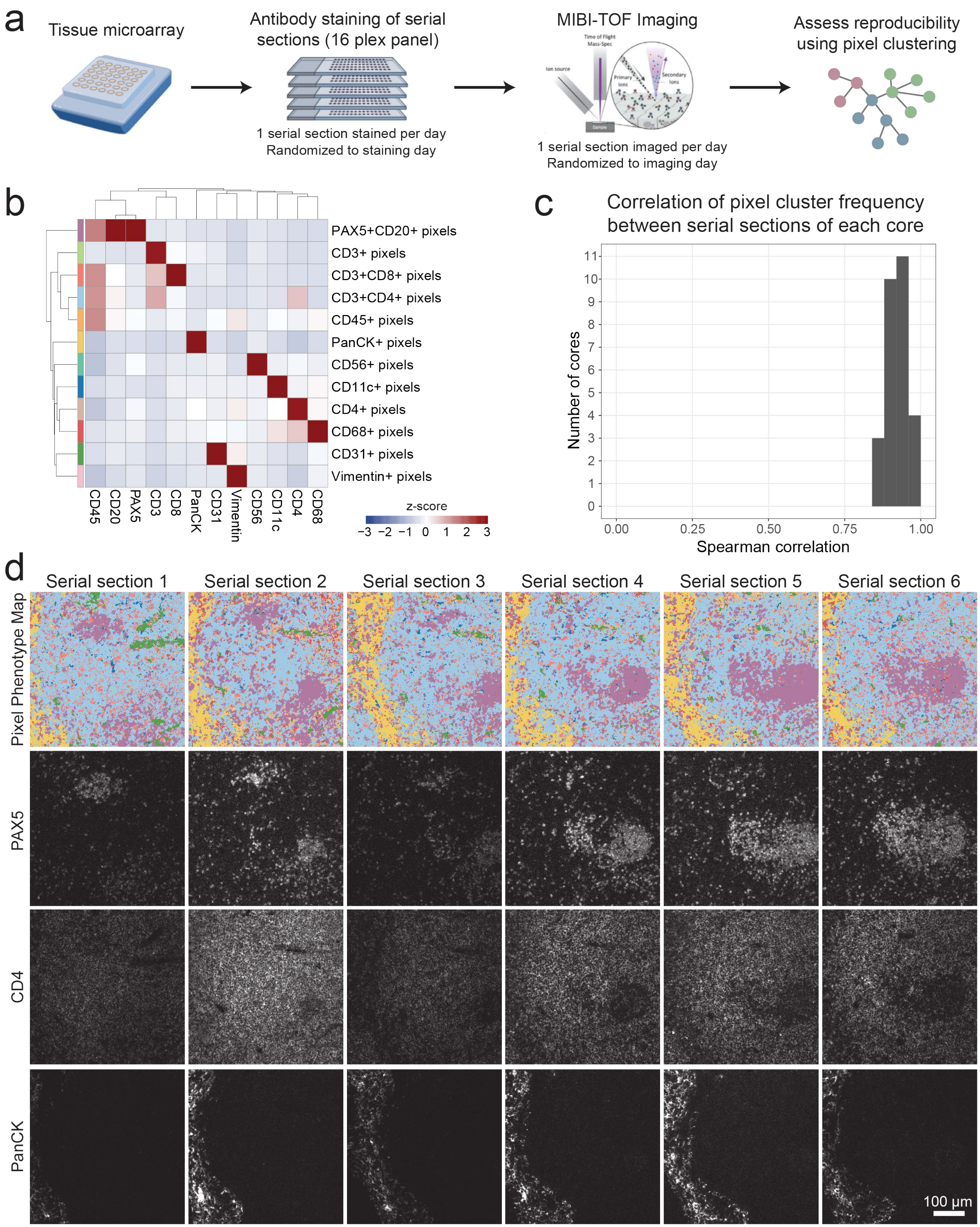

(A) A tissue microarray (TMA) comprised of various tissue types was serially sectioned, stained with a 16-plex panel, and imaged using MIBI-TOF.[35] The order that each serial section was stained and imaged was randomized. We then assessed the reproducibility of MIBI-TOF and pixel clustering by quantifying features between serial sections of the same TMA core. (B) Heatmap of pixel cluster phenotypes across the entire dataset. Expression values were z-scored for each marker. (C) The Spearman correlation between all serial sections of each TMA core using the frequency of pixel clusters in each FOV. (D) Example of pixel phenotype maps (colored according to the pixel clusters shown in B) and single-channel images for six serial sections of the same tonsil tissue core. The single-channel images have the same maximum value.

Supplementary Figure 15: Quantification of pixel cluster phenotypes in human hippocampus

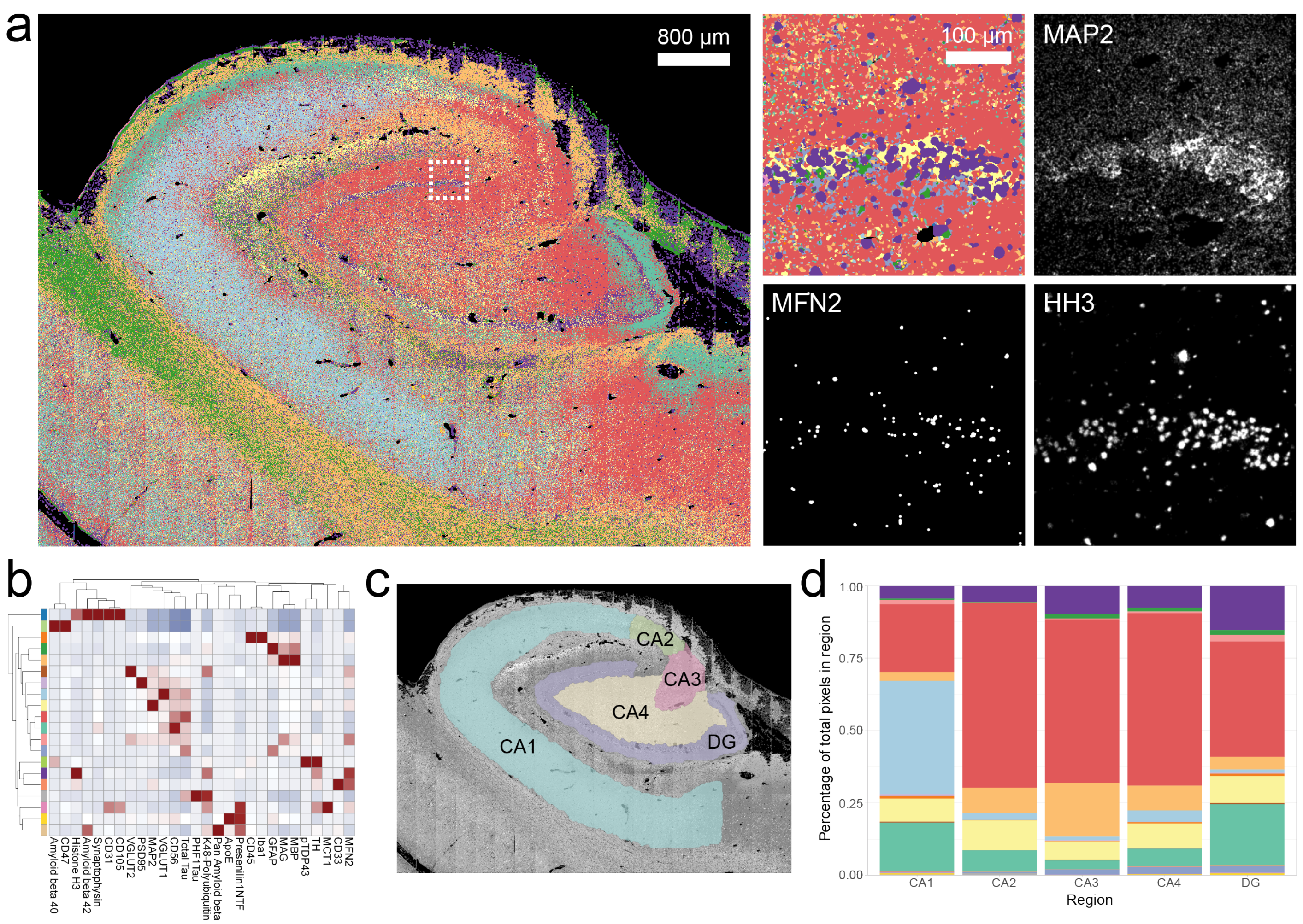

(A) Pixel phenotype map of MIBI-TOF data of cognitively normal human hippocampus tissue section[13]. 196 FOVs of 400 μm x 400 μm were captured by MIBI-TOF and tiled together. Insets (right) show local structure of the dentate gyrus, reflecting neuronal soma phenotypes as defined by MAP2, Histone H3, and MFN2 (mitofusion 2) expression. Total tiled MIBI-TOF image contained 63,642,954 non-zero pixels. (B) Heatmap of mean marker expression of pixel cluster phenotypes. Expression values were z-scored for each marker. Colors in the color bar correspond to the overlay in A. Proteins used for clustering include markers for microglia (CD45, Iba1) , astrocytes (GFAP), neurons (CD47, MAP2, TH, Tau, Synaptophysin, VGLUT1, VGLUT2, CD56), oligodendrocytes (MAG, MBP), vasculature (CD31, CD105, MCT1), proteopathy (Amyloid beta 40, Amyloid beta 42, PHF1Tau, Presenilin1NTF, pTDP43) and additional functional markers (Histone H3, MFN2, polyubiquitin 48, ApoE, CD33). (C) Hippocampus neuroanatomy as outlined by an expert neuropathologist. Dentate Gyrus (DG) and Cornu Ammonis (CA) regions 1-4 labelled. (D) Quantification of the pixel clusters belonging to each hippocampal region.

Supplementary Figure 16: Cell clustering using pixel composition in Pixie

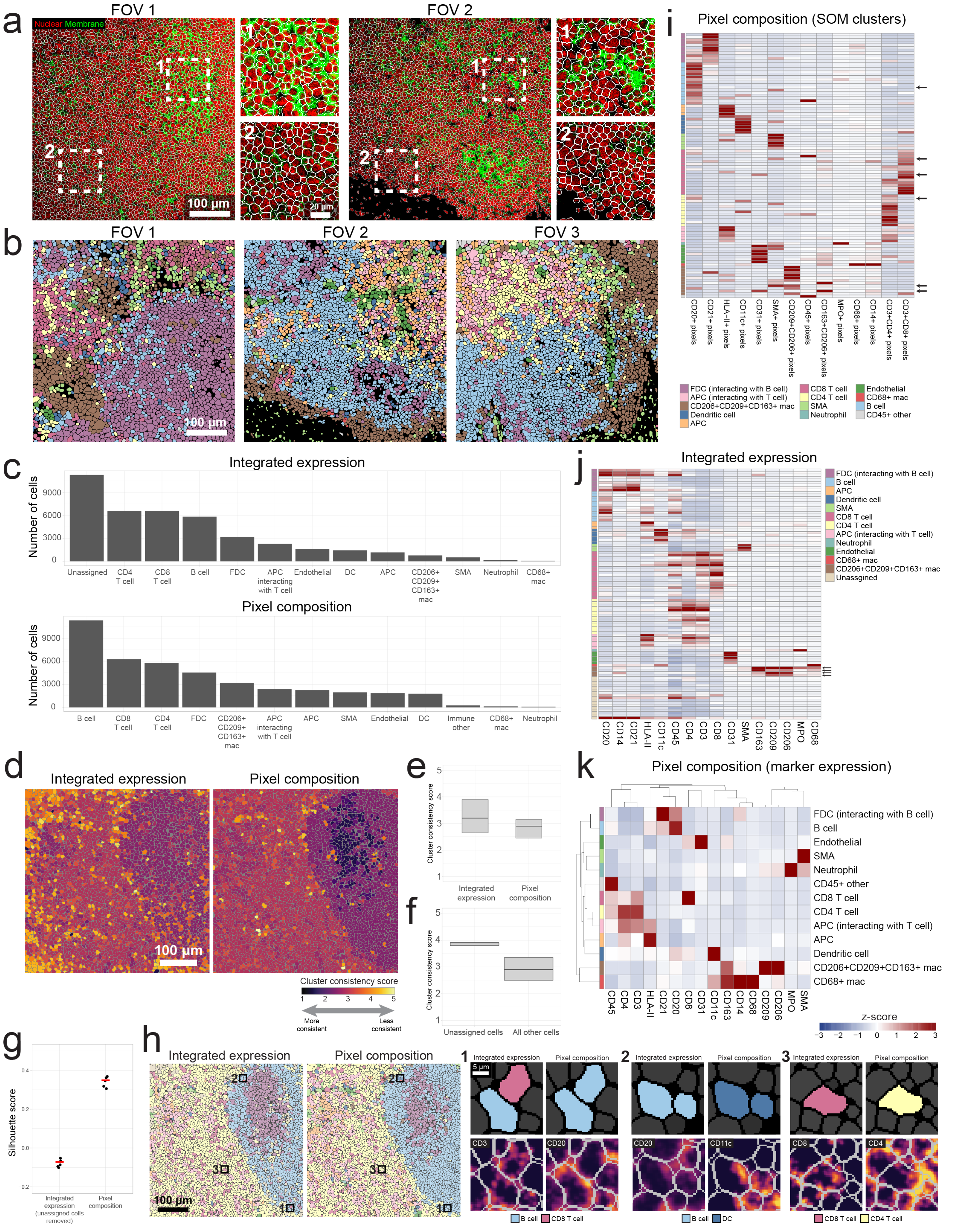

(A) Examples of segmentation quality. Images were segmented using the pre-trained Mesmer network (see Methods). We used histone H3 as the nuclear marker, and a combination of CD45, CD20, and HLA-II as the membrane marker. (B) Additional examples of cell phenotype maps for representative FOVs where cells were clustered using pixel cluster composition. Colors in the cell phenotype maps correspond to the heatmap in Fig. 5c. (C) Total number of cells of each phenotype identified using integrated expression (top) or pixel composition (bottom). (D) The FOV shown in Fig. 5e colored according to cluster consistency score, for clustering using integrated expression (left) or pixel composition (right). (E) Comparison of cluster consistency score for cell clusters obtained using integrated expression or pixel composition. Boxplots show median as the center and 25th and 75th percentiles as the bounds of the box. n=41,646 cells from 12 images. (F) For clustering using integrated expression, comparison of cluster consistency score for cells assigned to the unassigned group versus all other phenotypes. Boxplots show median as the center and 25th and 75th percentiles as the bounds of the box. n=41,493 cells from 12 images. (G) Silhouette score comparison between cell clusters obtained using integrated expression, where cells in the "Unassigned" cluster were removed, and cell clusters obtained using pixel composition. n=41,646 cells from 12 images. (H) Cell phenotype maps of the FOV shown in Fig. 5e (left) and examples where the clustering was ambiguous or incorrect. (I) Heatmap of the 100 SOM clusters, clustered using pixel composition, grouped according to their final annotation. Expression values were z-scored for each marker. Arrows on the right correspond to clusters that had ambiguous expression patterns that were manually inspected. (J) Heatmap of the 100 SOM clusters, clustered using integrated expression, grouped according to their final annotation. Expression values were z-scored for each marker. The arrows correspond to the CD206+ CD209+ CD163+ cluster, showing that all the individual clusters expressed the three markers with a z-score > 0. (K) Heatmap of marker expression for the cell phenotypes found using pixel composition. Marker expression was found by multiplying the number of each pixel cluster in each cell by the pixel cluster expression profile, then averaging across cells in the cluster. Expression values were z-scored for each marker.

Supplementary Figure 17: Cell clustering using integrated expression from pre-processed pixel data

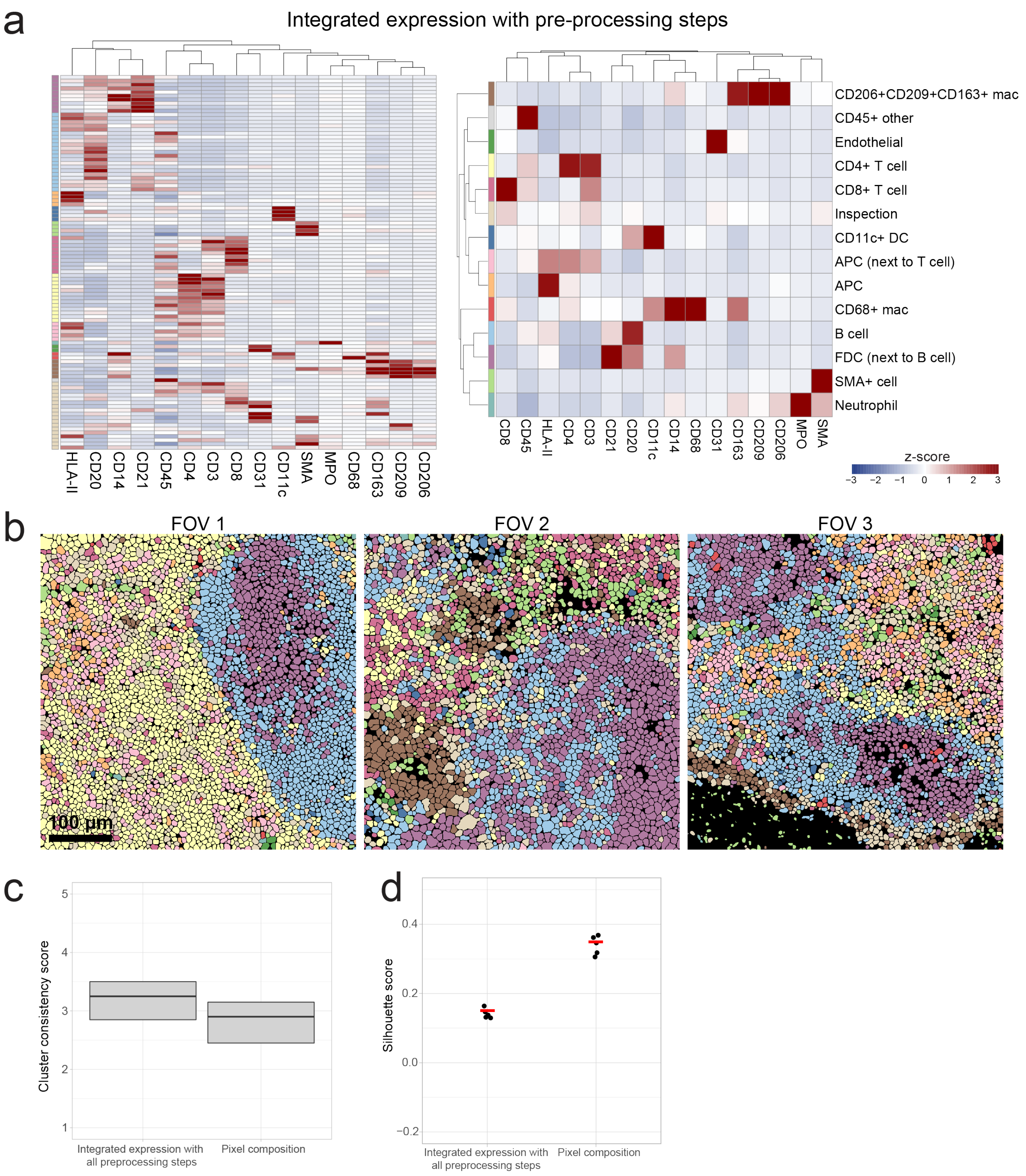

(A) Heatmap of the 100 SOM clusters (left) and annotated pixel cluster phenotypes (right) clustered using integrated expression, where the images were pre-processed as described for pixel clustering (i.e. Gaussian blur, pixel normalization, 99.9% normalization). Expression values were z-scored for each marker. (B) Cell phenotype maps for representative FOVs.

Colors correspond to the heatmaps in A. (C) Comparison of cluster consistency score between clustering using integrated expression of pre-processed pixel data or clustering using pixel composition. Boxplots show median as the center and 25th and 75th percentiles as the bounds of the box. n=41,646 cells from 12 images. (D) Comparison of Silhouette score between clustering using integrated expression of pre-processed pixel data or clustering using pixel composition. n=41,646 cells from 12 images.

Supplementary Figure 18: Cell clustering using segmentation from Ilastik/CellProfiler

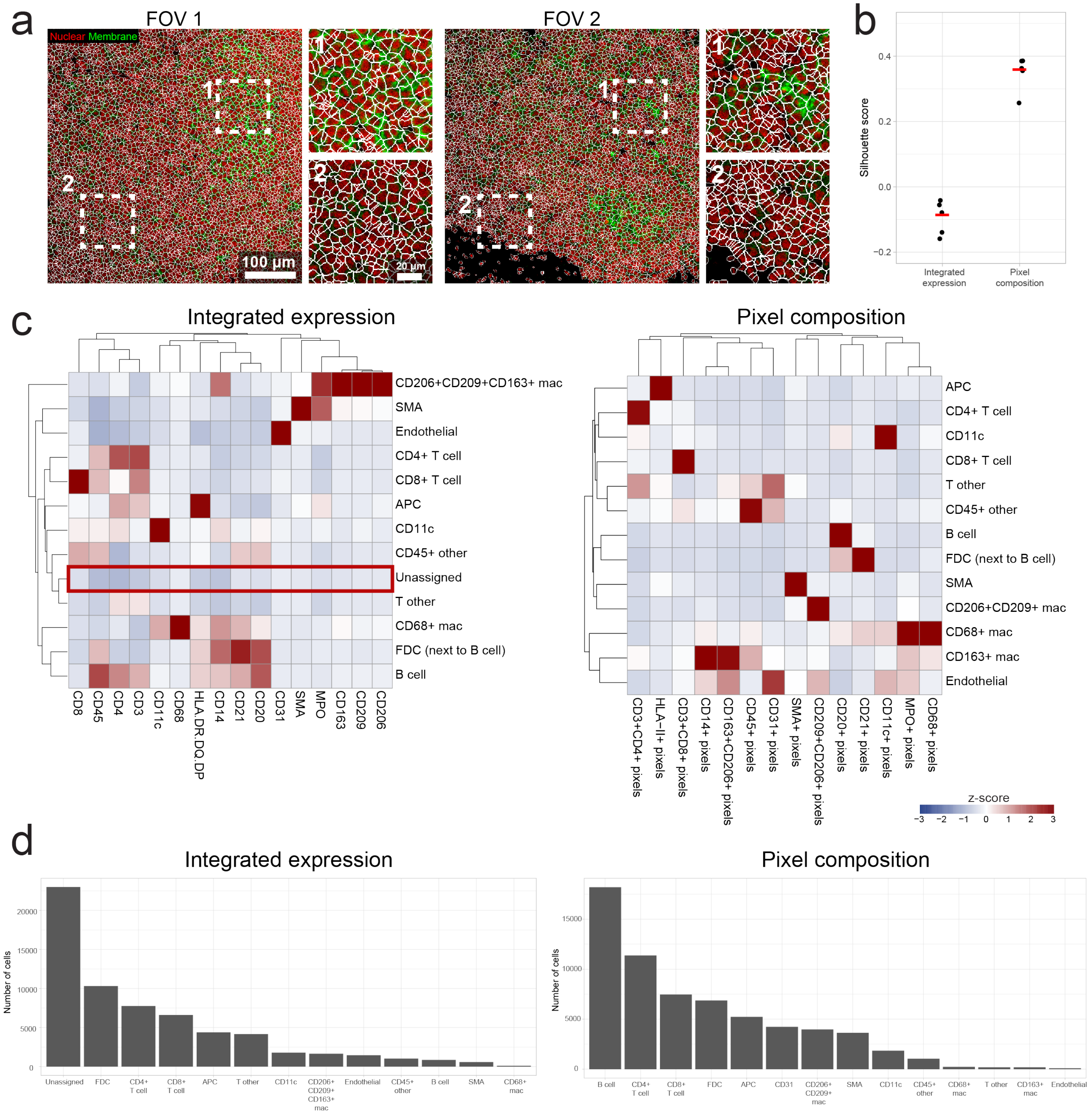

(A) Images showing segmentation performance using the Ilastik and CellProfiler segmentation pipeline.[52] We used histone H3 as the nuclear marker, and a combination of CD45, CD20, and HLA-II as the membrane marker. (B) Comparison of Silhouette score between pixel clustering using integrated expression or pixel composition, using the Ilastik/CellProfiler segmentation masks. n=63,549 cells from 12 images. (C) Heatmap of mean marker expression of cell cluster phenotypes using integrated expression (left) or pixel composition (right). Expression values were z-scored for each marker. (D) Total number of cells of each phenotype identified using integrated expression (left) or pixel composition (right).

Supplementary Figure 19: Comparison of cell clustering using manually labeled dataset

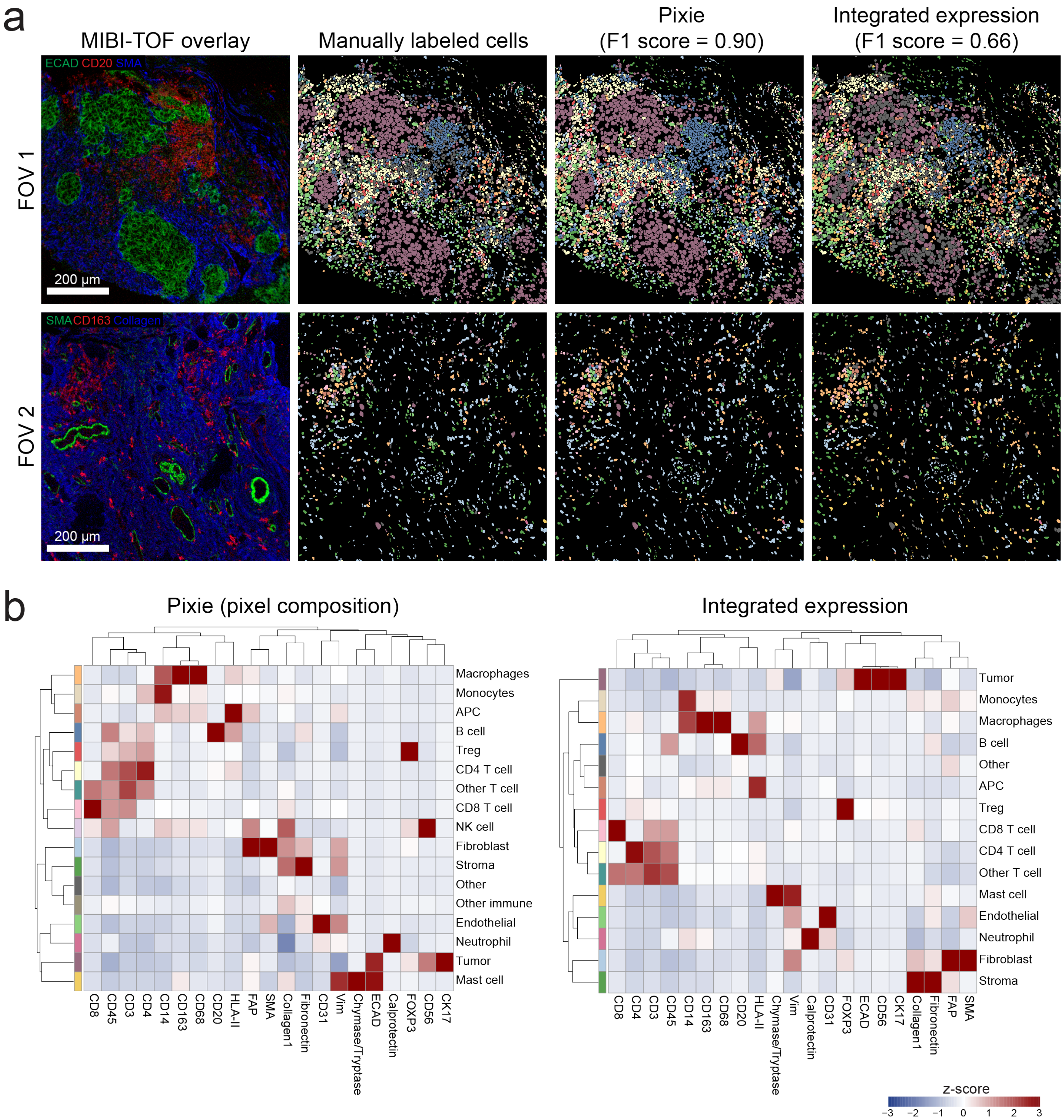

(A) Individual cells were manually annotated in MIBI-TOF images and used to assess cell clustering in Pixie. MIBI-TOF overlay (left) and cell phenotype maps (right) where cells are colored by their human-labeled phenotypes, where cells were clustered using pixel composition in Pixie, and where cells were clustered using integrated expression. (B) Heatmap of mean marker expression of the cell phenotypes identified using pixel composition (left) or integrated expression (right). Expression values were z-scored for each marker. The colors in the color bar correspond to the cell phenotype maps in A.

Supplementary Figure 20: Comparison of Pixie with Otsu thresholding

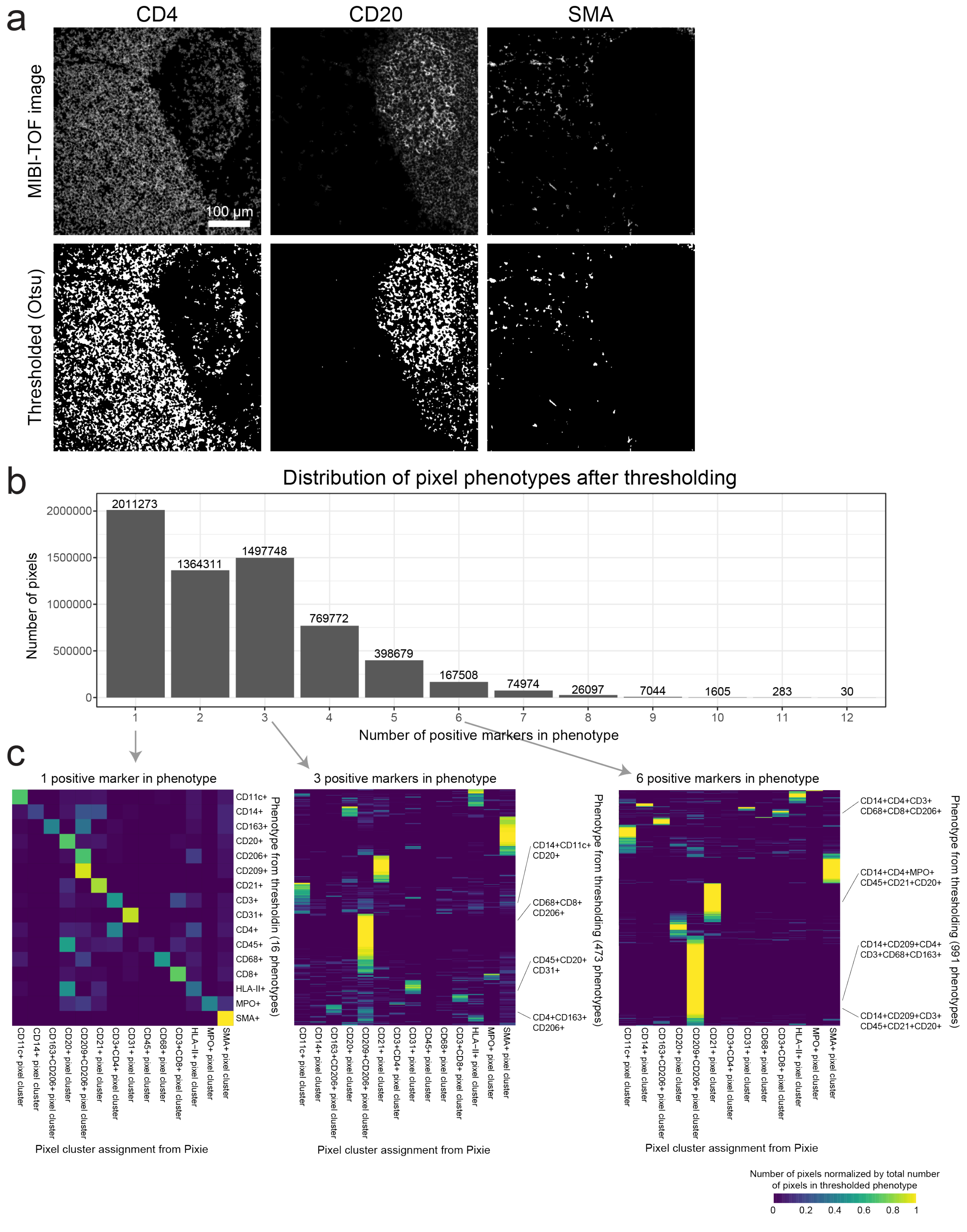

(A) Single-channel MIBI-TOF images (top row) were thresholded using Otsu's method to determine marker positivity. The thresholded images are shown in the bottom row (positive pixels are in white). Representative markers are shown. (B) For each pixel, we determined the number of markers that were called as positive using Otsu's method. Here, we are showing the distribution of the number of positive markers per pixel for the entire dataset. (C) Three representative examples showing the breakdown of the Otsu thresholded data (y axis) compared to the Pixie assignment (x axis). The heatmaps show the number of pixels normalized by the total number of pixels in the thresholded phenotype. For pixels that only contained 1 positive marker, there were 16 total phenotypes (i.e. the 16 markers included). For pixels that contained 3 positive markers, there were 473 total combinations, and for pixels that contained 6 positive markers, there were 991 total combinations.

Supplementary Figure 21: Reproducibility of cell clustering using pixel cluster composition

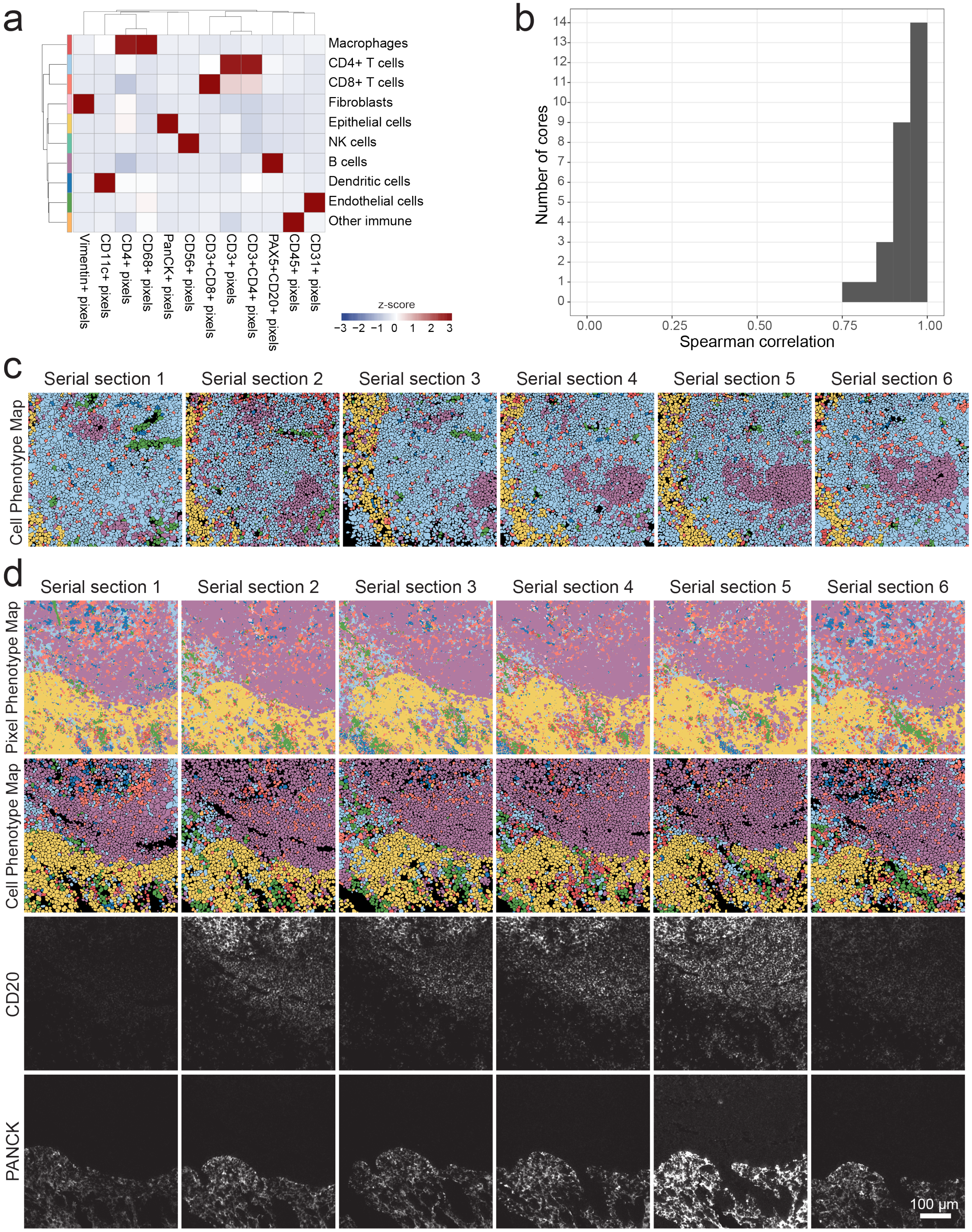

(A) Heatmap of cell phenotypes for the MIBI-TOF dataset in the reproducibility study shown in Supplementary Fig. 14.[35] Expression values were z-scored for each marker. (B) The Spearman correlation between all serial sections of each TMA core using the frequency of cell clusters in each FOV. (C) Cell phenotype maps (colored according to the cell phenotypes shown in A) for the tonsil tissue core shown in Supplementary Fig. 14d. (D) Additional example of pixel phenotype maps colored according to the pixel clusters shown in Supplementary Fig. 14 (top row), cell phenotype maps colored according to the cell clusters shown in A (second row), and single-channel images (third and fourth rows) for six serial sections of the same tonsil tissue core. The single-channel images have the same maximum value.

Supplementary Figure 22: Runtime analysis

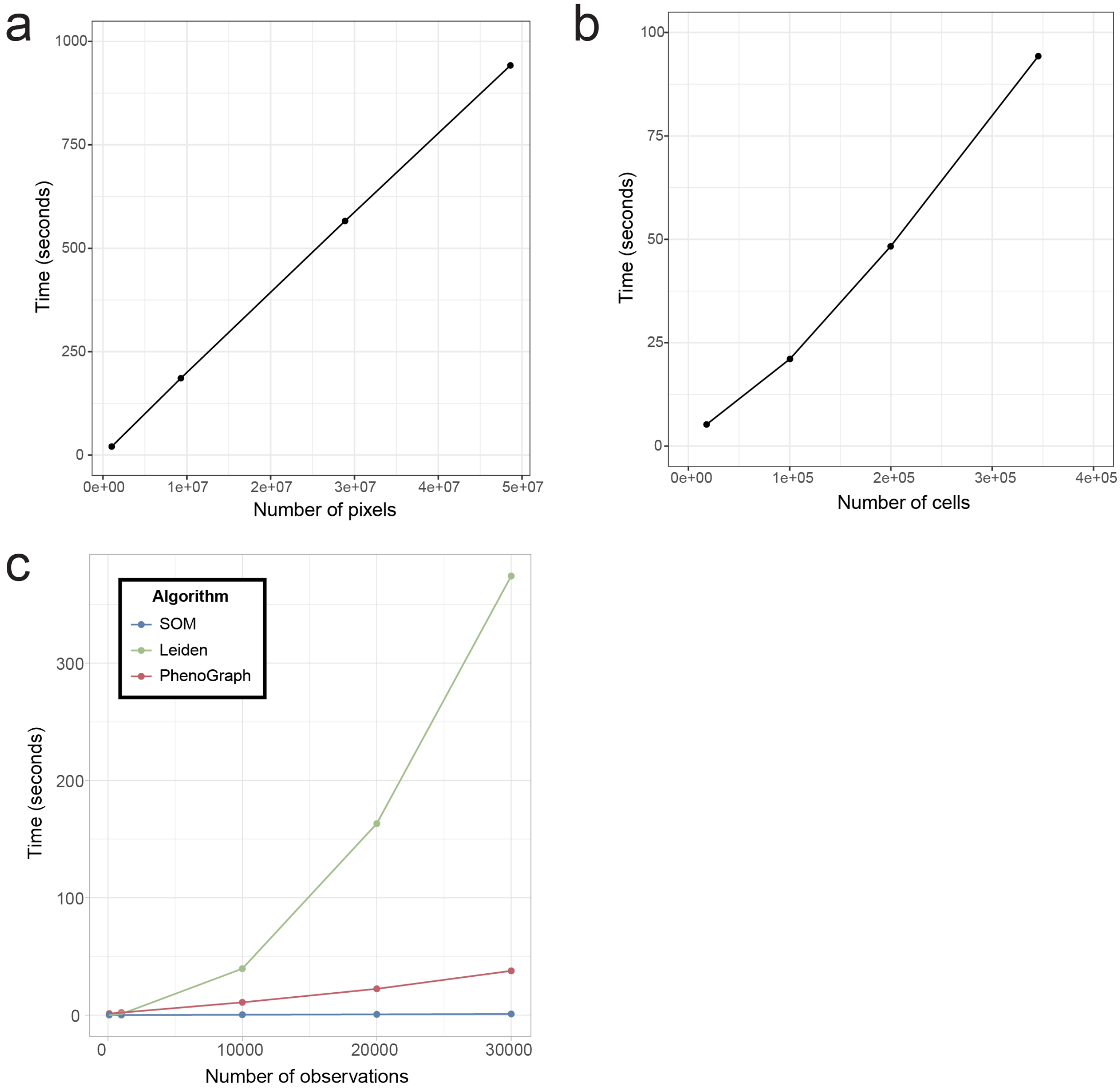

(A) Total Pixie runtime including all pre-processing steps and clustering for pixel clustering. (B) Total Pixie runtime including all pre-processing steps and clustering for cell clustering. (C) Runtime comparison between SOM (implemented in FlowSOM), Leiden (implemented in Seurat), and PhenoGraph (implemented in Rphenograph) clustering algorithms. Runtime comparison was performed on a Google Cloud Compute Engine instance with 16 vCPU and 128 GB of memory.

Supplementary Table 1: MIBI-TOF antibody staining panel for lymph node cohort

Target	Clone	Vendor	Catalog No.	Channel	Titer (ug/mL)	Day
Granzyme B	D6E9W	Cell Signaling	46890BF	141Pr	1.00	Day 1 (overnight)
Lag3	17B4	LSBio	LS-C18692	142Nd	1.00	Day 1 (overnight)
CD4	EPR6855	Abcam	ab181724	143Nd	0.50	Day 1 (overnight)
CD14	D7A2T	Cell Signaling	56082BF	144Nd	0.50	Day 1 (overnight)
Foxp3	236A/E7	BD Biosciences	624084	146Nd	1.00	Day 1 (overnight)
PD1	D4W2J	Cell Signaling	86163BF	147Sm	2.00	Day 1 (overnight)
CD31	EP3095	Abcam	ab216459	148Nd	0.50	Day 1 (overnight)
PD-L1-biotin	E1L3N	Cell Signaling	13684BF	149Sm	2.50	Day 1 (overnight)
CD21	SP186	Spring	M4864.C	150Nd	0.50	Day 1 (overnight)
Ki67	8D5	Cell Signaling	9449BF	151Eu	0.50	Day 1 (overnight)
CD209/DC-SIGN	DCN46	BD Biosciences	624084	152Sm	1.00	Day 1 (overnight)
CD206	685645	R&D Systems	MAB25341	153Eu	1.00	Day 1 (overnight)
pS6	D57.2.2E	Cell Signaling	4858BF	154Sm	0.50	Day 1 (overnight)
CD68	D4B9C	Cell Signaling	76437BF	156Gd	0.50	Day 1 (overnight)
Tbet	4B10	Abcam	ab91109	157Gd	2.00	Day 1 (overnight)
CD8	C8/144B	Cell Marque	108M-OEM1404	158Gd	0.50	Day 1 (overnight)
CD3	D7A6E	Cell Signaling	85061BF	159Tb	0.50	Day 1 (overnight)
IDO	SP260	Spring	M5604.C	160Gd	1.00	Day 1 (overnight)
CD11c	EP1347Y	Abcam	ab216655	161Dy	0.50	Day 1 (overnight)
TIM3	EPR22241	Abcam	ab242080	162Dy	2.00	Day 1 (overnight)
CD163	D6U1J	Cell Signaling	93498BF	163Dy	2.00	Day 1 (overnight)
CD20	L26	Cell Marque	120M-8-OEM	164Er	0.50	Day 1 (overnight)
CD16	D1N9L	Cell Signaling	24326BF	165Ho	1.00	Day 1 (overnight)
GLUT1	EPR3915	Abcam	ab196357	166Er	0.50	Day 1 (overnight)
HLA-DR	EPR3692	Abcam	ab208650	167Er	0.50	Day 1 (overnight)
CD57	NK/804	Abcam	ab212408	168Er	0.50	Day 1 (overnight)
CD45	D9M8I	Cell Signaling	13917BF	169Tm	0.75	Day 1 (overnight)
CD45RO	UCHL1	BioLegend	304202	171YB	1.00	Day 1 (overnight)
CD138	EPR6454	Abcam	ab216458	173Yb	1.00	Day 1 (overnight)
MPO	polyclonal	R&D Systems	AF3667	174Yb	1.00	Day 1 (overnight)
Vimentin	D21H3	Cell Signaling	5741BF	113In	4.00	Day 2 (1 hr)
SMA	SP171	Cell Signaling	19245BF	115In	4.00	Day 2 (1 hr)
Biotin	1D4-C5	BioLegend	409002	149Sm	4.00	Day 2 (1 hr)
H3K9Ac	C5B11	Cell Signaling	9649BF	170Er	2.00	Day 2 (1 hr)
H3K27me3	C36B11	Cell Signaling	9733BF	172Yb	2.00	Day 2 (1 hr)
Tryptase	794	Abcam	ab212156	176Yb	0.25	Day 2 (1 hr)
HH3	D1H2	Cell Signaling	4499BF	89Y	4.00	Day 2 (1 hr)

Antibodies were combined into one panel and stained overnight (Day 1) or for 1 hour (Day 2). The antibody clone, vendor, catalog number, mass channel, conjugated metal, and titer used in the final panel are included.
