# Supplementary material for: Robust phenotyping of highly multiplexed tissue imaging data using pixel-level clustering
Source: Nat Commun. 2023 Aug 1;14:4618. doi: 10.1038/s41467-023-40068-5 (PMC10393943; doi:10.1038/s41467-023-40068-5)
Supplement: Supplementary file 3 — Reporting Summary [file 41467_2023_40068_MOESM3_ESM.pdf]

nature portfolio

Corresponding author(s): Michael Angelo

Last updated by author(s): Jul 5, 2023

Reporting Summary

Nature Portfolio wishes to improve the reproducibility of the work that we publish. This form provides structure for consistency and transparency in reporting. For further information on Nature Portfolio policies, see our Editorial Policies and the Editorial Policy Checklist.

Statistics

For all statistical analyses, confirm that the following items are present in the figure legend, table legend, main text, or Methods section.

n/a	Confirmed	
[ ]	[x]	The exact sample size (*n*) for each experimental group/condition, given as a discrete number and unit of measurement
[ ]	[x]	A statement on whether measurements were taken from distinct samples or whether the same sample was measured repeatedly
[ ]	[x]	The statistical test(s) used AND whether they are one- or two-sided<br>*Only common tests should be described solely by name; describe more complex techniques in the Methods section.*
[x]	[ ]	A description of all covariates tested
[x]	[ ]	A description of any assumptions or corrections, such as tests of normality and adjustment for multiple comparisons
[ ]	[x]	A full description of the statistical parameters including central tendency (e.g. means) or other basic estimates (e.g. regression coefficient) AND variation (e.g. standard deviation) or associated estimates of uncertainty (e.g. confidence intervals)
[ ]	[x]	For null hypothesis testing, the test statistic (e.g. *F*, *t*, *r*) with confidence intervals, effect sizes, degrees of freedom and *P* value noted<br>*Give P values as exact values whenever suitable.*
[x]	[ ]	For Bayesian analysis, information on the choice of priors and Markov chain Monte Carlo settings
[x]	[ ]	For hierarchical and complex designs, identification of the appropriate level for tests and full reporting of outcomes
[ ]	[x]	Estimates of effect sizes (e.g. Cohen's *d*, Pearson's *r*), indicating how they were calculated

Our web collection on statistics for biologists contains articles on many of the points above.

Software and code

Policy information about availability of computer code

Data collection	No software was used for data collection.
Data analysis	R (4.1.2), FlowSOM (1.21.0); ConsensusClusterPlus (1.58.0), ggplot2 (3.3.5), data.table (1.14.2), gridExtra (2.3), pheatmap (1.0.12), viridis (0.6.2), dplyr (1.0.7), arrow (7.0.0), ggpubr (0.4.0), flowCore(2.6.0), patchwork (1.1.1), Seurat (4.0.6), Rphenograph (0.99.1); Python (3.8.13), pandas (1.5.3), numpy (1.23.5), matplotlib (3.7.1), PIL (9.5.0), scipy (1.10.1), skimage (0.19.3), feather (0.4.1)<br><br>User-friendly Jupyter notebooks for running Pixie are available at https://github.com/angelolab/pixie. The code used to generate the figures is available at https://github.com/angelolab/publications/tree/main/2022-Liu_etal_Pixie and also deposited on Zenodo at https://doi.org/10.5281/zenodo.8097933.65.

For manuscripts utilizing custom algorithms or software that are central to the research but not yet described in published literature, software must be made available to editors and reviewers. We strongly encourage code deposition in a community repository (e.g. GitHub). See the Nature Portfolio guidelines for submitting code & software for further information.

Data

Policy information about availability of data

All manuscripts must include a data availability statement. This statement should provide the following information, where applicable:

- Accession codes, unique identifiers, or web links for publicly available datasets
- A description of any restrictions on data availability
- For clinical datasets or third party data, please ensure that the statement adheres to our policy

The data generated in this study and source data have been deposited in Zenodo under accession code https://doi.org/10.5281/zenodo.8118725. Publicly available datasets used in this study are cited in the text. TNBC data will be published in a forthcoming manuscript and will be shared upon reasonable request to the corresponding author. Requests will be fulfilled within a week.

Human research participants

Policy information about studies involving human research participants and Sex and Gender in Research.

Reporting on sex and gender	Data was obtained from previous publications. See previous publications.
Population characteristics	Data was obtained from previous publications. See previous publications.
Recruitment	Data was obtained from previous publications. See previous publications.
Ethics oversight	Data was obtained from previous publications. See previous publications.

Note that full information on the approval of the study protocol must also be provided in the manuscript.

Field-specific reporting

Please select the one below that is the best fit for your research. If you are not sure, read the appropriate sections before making your selection.

[x] Life sciences [ ] Behavioural & social sciences [ ] Ecological, evolutionary & environmental sciences

For a reference copy of the document with all sections, see nature.com/documents/nr-reporting-summary-flat.pdf

Life sciences study design

All studies must disclose on these points even when the disclosure is negative.

Sample size	Data was obtained from previous publications. We used 10 different datasets across 4 different imaging technologies. We chose datasets to cover a variety of tissue types and disease states to sufficiently show the applicability of this method.
Data exclusions	No exclusions.
Replication	Biological samples were not replicated in this study. We replicated our algorithm on the same datasets to assess the inherent stochasticity of the algorithm. Setting a random seed allowed us to successfully replicate our results.
Randomization	Randomization is not relevant to this study since we are not comparing between experimental groups.
Blinding	Blinding is not relevant to this study because we are not considering clinical outcomes.

Reporting for specific materials, systems and methods

We require information from authors about some types of materials, experimental systems and methods used in many studies. Here, indicate whether each material, system or method listed is relevant to your study. If you are not sure if a list item applies to your research, read the appropriate section before selecting a response.

Materials & experimental systems

n/a	Involved in the study	
☐	☒	Antibodies
☒	☐	Eukaryotic cell lines
☒	☐	Palaeontology and archaeology
☒	☐	Animals and other organisms
☒	☐	Clinical data
☒	☐	Dual use research of concern

Methods

n/a	Involved in the study	
☒	☐	ChIP-seq
☒	☐	Flow cytometry
☒	☐	MRI-based neuroimaging

Antibodies

Antibodies used

Granzyme B (D6E9W)
Lag3 (17B4)
CD4 (EPR6855)
CD14 (D7A2T)
CC3 (Asp175)
Foxp3 (236A/E7)
PD1 (D4W2J)
CD31 (EP3095)
PD-L1-biotin (E1L3N)
CD21 (SP186)
Ki67 (8D5)
CD209/DC-SIGN (DCN46)
CD206 (685645)
pS6 (D57.2.2E)
CD68 (D4B9C)
Tbet (4B10)
CD8 (C8/144B)
CD3 (D7A6E)
IDO (SP260)
CD11c (EP1347Y)
TIM3 (EPR22241)
CD163 (D6U1J)
CD20 (L26)
CD16 (D1N9L)
GLUT1 (EPR3915)
HLA-DR (EPR3692)
CD57 (NK/804)
CD45 (D9M8I)
CD45RO (UCHL1)
CD138 (EPR6454)
MPO (polyclonal)
Vimentin (D21H3)
SMA (SP171)
biotin (1D4-C5)
H3K9Ac (C5B11)
H3K27me3 (C36B11)
Tryptase (794)
HH3 (D1H2)

Validation

All antibodies were validated using chromogenic immunohistochemisry on control tissues, including lymph node and tonsil. Antibodies and validation for previously published studies are available at the respective publications.
