# Supplementary material for: Robust phenotyping of highly multiplexed tissue imaging data using pixel-level clustering
Source: Nat Commun. 2023 Aug 1;14:4618. doi: 10.1038/s41467-023-40068-5 (PMC10393943; doi:10.1038/s41467-023-40068-5)
Supplement: Supplementary file 4 — Description of Additional Supplementary Files [file 41467_2023_40068_MOESM4_ESM.pdf]

Description of Additional Supplementary Files

Supplementary Movie 1
Demonstration of the graphical user interface (GUI) for manual metacluster adjustment and annotation, publicly available on Github.
