# Supplementary material for: Robust phenotyping of highly multiplexed tissue imaging data using pixel-level clustering
Source: Nat Commun. 2023 Aug 1;14:4618. doi: 10.1038/s41467-023-40068-5 (PMC10393943; doi:10.1038/s41467-023-40068-5)
Supplement: Supplementary file 2 — Peer Review File [file 41467_2023_40068_MOESM2_ESM.pdf]

nature portfolio

Peer Review File

Robust phenotyping of highly multiplexed tissue imaging data using pixel-level clustering

Open Access This file is licensed under a Creative Commons Attribution 4.0 International License, which permits use, sharing, adaptation, distribution and reproduction in any medium or format, as long as you give appropriate credit to the original author(s) and the source, provide a link to the Creative Commons license, and indicate if changes were made. In the cases where the authors are anonymous, such as is the case for the reports of anonymous peer reviewers, author attribution should be to 'Anonymous Referee' followed by a clear attribution to the source work. The images or other third party material in this file are included in the article's Creative Commons license, unless indicated otherwise in a credit line to the material. If material is not included in the article's Creative Commons license and your intended use is not permitted by statutory regulation or exceeds the permitted use, you will need to obtain permission directly from the copyright holder. To view a copy of this license, visit http://creativecommons.org/licenses/by/4.0/.

This manuscript has been previously reviewed at another journal that is not operating a transparent peer review scheme. This document only contains reviewer comments and rebuttal letters for versions considered at *Nature Communications*.

REVIEWERS' COMMENTS

Reviewer #1 (Remarks to the Author):

The authors have done a thorough response to the reviewer comments and I have no further requests.

Reviewer #2 (Remarks to the Author):

I would like to express my gratitude to the reviewers for incorporating my suggestions into their manuscript. I also want to commend the team for their dedicated efforts in creating a pip-installable library and crafting the illustrative Jupyter notebooks. Undoubtedly, these endeavors will enhance the method's accessibility and increase its usefulness to the broader scientific community. I found the entire process to be straightforward and seamless, and I wish the team all the best in their future endeavors.

Reviewer #3 (Remarks to the Author):

The authors have sufficiently addressed my concerns during the revision of the manuscript.
